# Which halogen to choose? Comparing the effects of chlorine and fluorine as bioisosteric substituents in drug design

**DOI:** 10.1039/d5sc07348k

**Published:** 2026-01-08

**Authors:** Connor J. E. Summerfield, Graham Pattison

**Affiliations:** a Department of Chemistry, School of Natural Sciences, University of Lincoln, Joseph Banks Laboratories Green Lane Lincoln UK gpattison@lincoln.ac.uk

## Abstract

The effects of fluorine and chlorine on pharmaceutical systems are compared using a molecular matched pair analysis. Effects on binding constants, physicochemical properties such as lipophilicity and solubility, as well as on metabolic properties will show the differences and similarities between the elements in a medicinal context. Factors such as the difference in electronegativity, polarizability, hydrogen-bond acceptor ability, as well as conformational effects are discussed to put the differences between the elements into context, using real case studies taken from the literature.

## Abstract 2

We use a molecular matched pair analysis to compare the properties of drug compounds containing fluorine and chlorine, looking at and explaining similarities and differences in properties and highlighting these using real examples.

## Graphical abstract

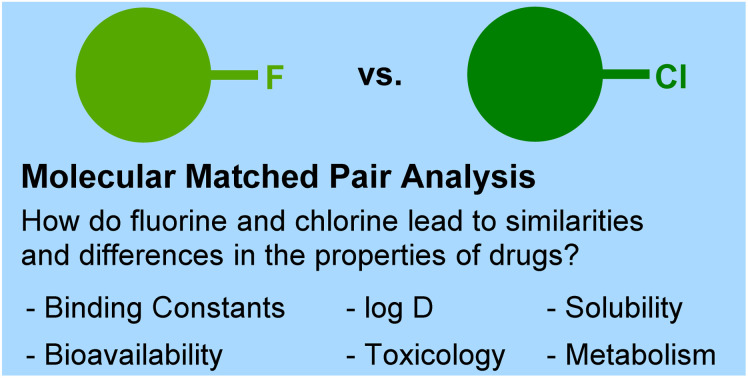

## Introduction

The halogens have proven especially influential in the design of drugs. An analysis of the top 200 small molecule drugs by retail sales in 2023 showed 59 (30%) contained at least one fluorine atom, and 30 (15%) contained at least one chlorine atom.^1^ In particular, when used as substituents on aromatic rings, chlorine and fluorine are widespread in their application.^2^

**Fig. 1:**
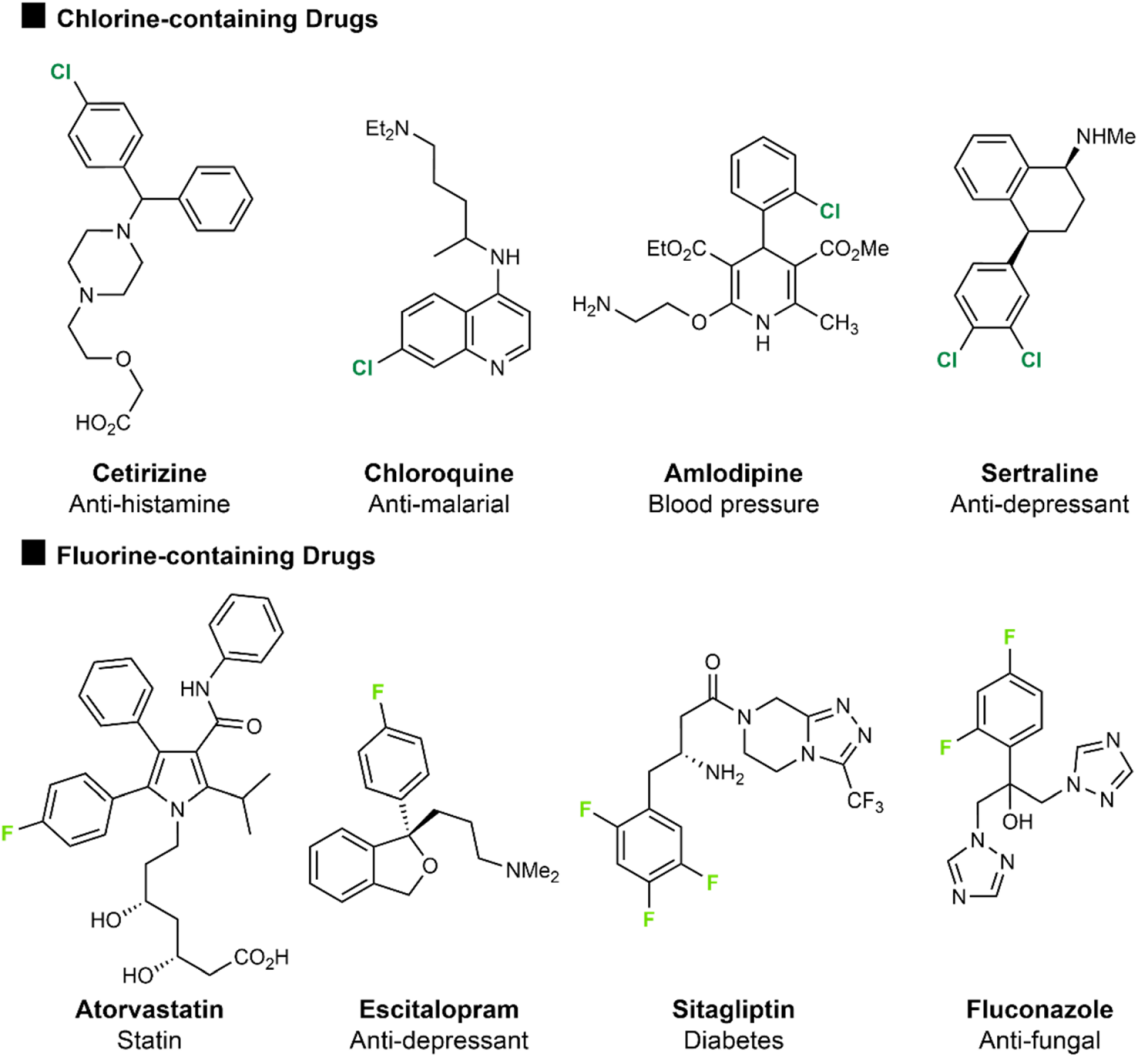
Structures of chlorinated and fluorinated drugs.

Fig. 1 presents a series of approved and marketed drug compounds containing chlorine or fluorine, demonstrating how these two halogens have been broadly applicable across a range of drug application areas.^3^ Of these drugs atorvastatin (Lipitor), a statin treatment for high cholesterol containing an aryl fluorine atom, was the most prescribed drug in 2022. Amlodipine, the 5^th^ most prescribed drug in 2022, is a treatment for high blood pressure and contains an *ortho*-chlorine substituent on an aromatic ring. Sertraline (11^th^ most prescribed in 2022) is an SSRI used to treat depression and anxiety and features two chlorine atoms on an aromatic ring. Similarly, escitalopram (15^th^ most prescribed in 2022) is also an SSRI anti-depressant but contains a *para*-fluorine substituent on an aromatic ring. The presence of both fluorine and chlorine in the top 200 drugs by retail sales has remained consistent over time since 2006, with 15–18% containing at least one fluorine atom and 9–15% containing chlorine, despite the increasing prevalence of biologic drugs which has reduced the proportion of small molecule drugs on the market overall. This likely effectively presents an increase in the number of small-molecule drugs containing a halogen atom.

Fluorine and chlorine have been used to influence a wide variety of medicinal properties, ranging from modifying binding to targets to improving pharmacokinetics, bioavailability or metabolic stability. Although the properties of these two halogen atoms are inherently similar in many ways, they do differ and can lead to drug compounds with different properties in many cases. However, at the outset of a structure–property optimization programme it is not always clear which of these two halogens will offer the biggest improvement in a desired property. This review article aims to describe and to understand the key similarities and differences between the two halogens across a range of pharmaceutical properties, including physical and chemical properties, binding to targets, pharmacokinetics and metabolic properties. We hope this will help practitioners to decide which halogen could be most useful in their system of study. The use of both chlorine^4^ and fluorine^5^ in medicinal chemistry have been reviewed previously, but to the best of our knowledge a comparative analysis of the two halogens has not been previously made.

To understand the similarities and differences between chlorine and fluorine better a Molecular Matched Pair (MMP) Analysis^6^ was undertaken to compare the performance of these two halogen atoms across a range of pharmaceutical properties. MMP analysis compares a pair of molecules in which the two molecules differ in structure only at a single position. In this case therefore the only difference between a matched pair is that one compound contains a fluorine atom, and its matched pair has a chlorine atom at a same specific site in the molecule, with no other structural differences. As both *para*-chlorine and fluorine substituents often feature in early stages of a SAR optimization process,^7^ there should be many examples of chlorine/fluorine matched pairs available in the literature. There are also many synthetic routes to, and building blocks containing, these elements at a range of positions around aromatic and heteroaromatic rings. This will allow us to examine which of the two halogen atoms shows the best performance on average for a particular property, and throughout the review examples will be highlighted showing similarities and differences between the two halogens.

## Method

To perform this molecular matched pair analysis, data was downloaded from the ChEMBL database^8^ covering the years 2013–23 and the major medicinal chemistry journals *J. Med. Chem.*, *Bioorg. Med. Chem.* (+*Lett.*), *Eur. J. Med. Chem.*, *ACS Med. Chem. Lett.*, *MedChemComm* and *RSC Med. Chem.* This downloaded data was then curated using the filter function on Microsoft Excel to select particular medicinal chemistry parameters of interest (for example binding constants). The KNIME cheminformatics software package was then used to highlight and save molecular matched pairs containing either a fluorine or chlorine atom on a ring using the open access RDKit ‘MMP Molecule Fragment’ and ‘Fragments to MMPS’ nodes, setting fragmentation to only acyclic single bonds to rings. The data was then copied to Excel and checked within each pair that the ChEMBL Assay ID codes matched using the EXACT function, to confirm that data pairs were being compared under the same experimental conditions. An Excel spreadsheet containing the relevant data is made available as SI. This data is analysed and discussed below, and the results are clarified with relevant examples.

## Physical and chemical properties

An understanding of the differing physical and chemical properties of chlorinated and fluorinated molecules will provide the foundation for us to compare their behaviour in pharmaceuticals. Some of the key properties to understand include polarity and electronegativity, atomic size, as well as acid–base properties and these will be discussed in the sections below.

The position of fluorine and chlorine at the top of the halogens in the periodic table does give them many similarities and unique properties, but the smaller size of fluorine also leads to some key differences.^9^

Fluorine is the most electronegative of all the elements, with a Pauling electronegativity value of 3.98, compared to chlorine with a value of 3.16. This leads to a significant polar character to the carbon–halogen bond. In particular for fluorine this leads to a significant electrostatic character of the C–F bond which in turn leads to bond strengthening. The C–F bond is the strongest bond of any heteroatom to carbon with a mean bond dissociation energy of 105.4 kcal mol^−1^ compared to just 78.5 kcal mol^−1^ for the C–Cl bond. The C–F bond is also very short at 1.47 Å, compared to the C–Cl bond at 1.77 Å.

**Fig. 2:**
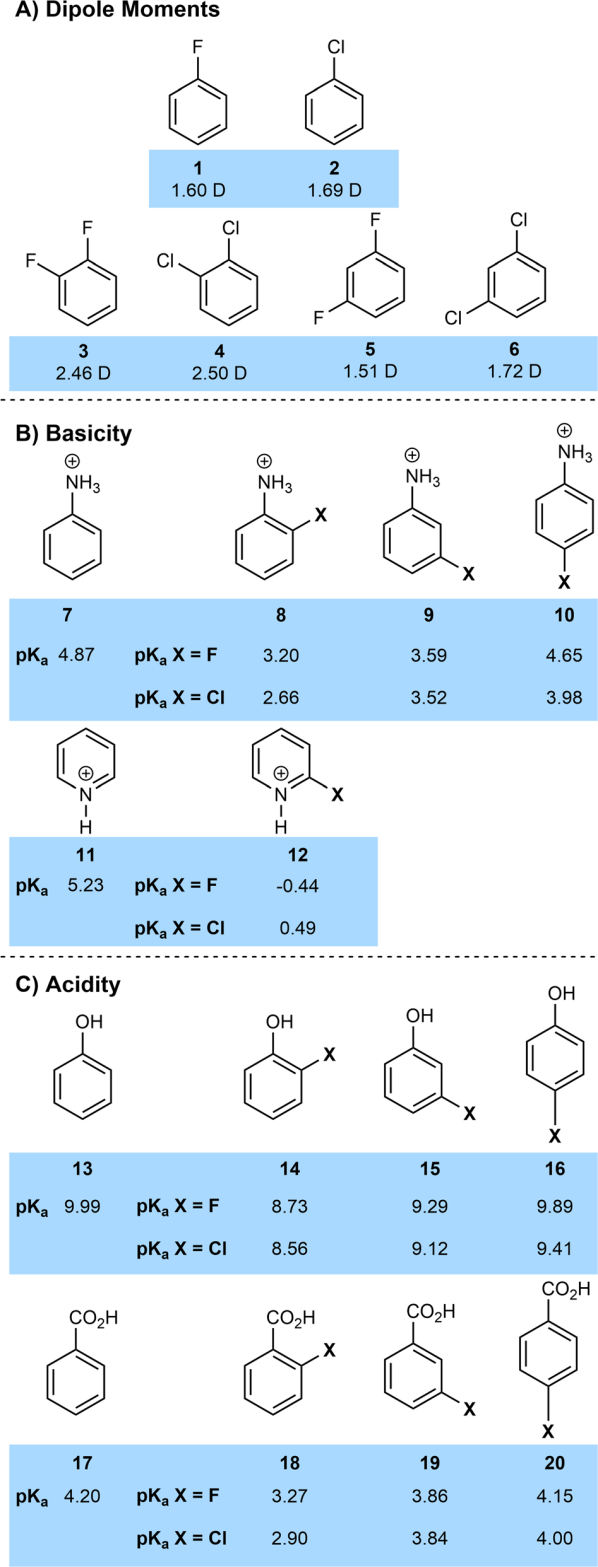
Comparison of physical properties of chlorinated and fluorinated compounds.

However, the dipole moments of fluorinated and chlorinated molecules are much closer in general than a cursory analysis of electronegativity might predict. Fluoromethane has a dipole moment of 1.85 D, compared to 1.87 D for chloromethane, the similar values being due to the competing effects of the increased charge separation of the C–F bond and the increased length of the C–Cl bond.^10^ A similar analysis can be performed on various halogenated benzenes (Fig. 2A) with fluorinated and chlorinated systems having very similar dipole moments and the dipoles of 1,2-dihalogenated arenes being particularly large.^11^

The small size of the fluorine atom is also emphasized by various steric parameters. The van der Waals radius of the fluorine atom is 1.47 Å, with a chlorine atom being larger at 1.74 Å.^12^ Fluorine is the closest steric match available for both a hydrogen atom (1.20 Å) and oxygen atom (1.42 Å), whilst a chlorine is a closer steric match for a methyl group.

The electron-withdrawing effect of the halogens has significant effects on acidity and basicity (Fig. 2B and C).^13^ In general, halogenated compounds are more acidic than their non-halogenated counterparts. This is largely an inductive effect so on an aromatic ring, this effect is normally most significant at the ortho positions and reduces with distance (*e.g.*8*vs.*7; 14*vs.*13; 18*vs.*17). Across anilines, phenols and benzoic acid the acidifying effect of chlorine is slightly greater than that of fluorine (*e.g.* p*K*_a_ 2-chlorobenzoic acid 18-Cl = 2.90; p*K*_a_ 2-fluorobenzoic acid 18-F = 3.27). This is because fluorine is a moderate π-donor, which slightly overcomes fluorine's greater electronegativity. Of course, this π-donor effect is not significant in aliphatic systems where fluorinated compounds are generally more acidic then chlorinated ones (for example the p*K*_a_ of trifluoroacetic acid is −0.25 and that of trichloroacetic acid is 0.65). Overall, the similarity of p*K*_a_ values for chlorinated and fluorinated compounds make it unlikely in most cases that a change in halogen substituent would lead to a significant difference in protonation state of a drug at physiological pH.

## Binding and activity

Inhibitor constants such as *K*_i_, IC_50_ and EC_50_ provide valuable information about the activity of a drug molecule. Information provided by the ChEMBL database allows us to separate these inhibitor constant values into assays which are classified as ‘Binding’ and those which are classified as ‘Functional’. This allowed us to probe deeper into whether any differences in inhibitor constants of chlorinated and fluorinated compounds are reflected across both binding events and observed functional bioactivity.

Values of *K*_i_, IC_50_ and EC_50_ were selected from the ChEMBL database then further separated into ‘Binding’ and ‘Functional’ codes. Matched pair analysis was performed on each sample separately.

**Fig. 3:**
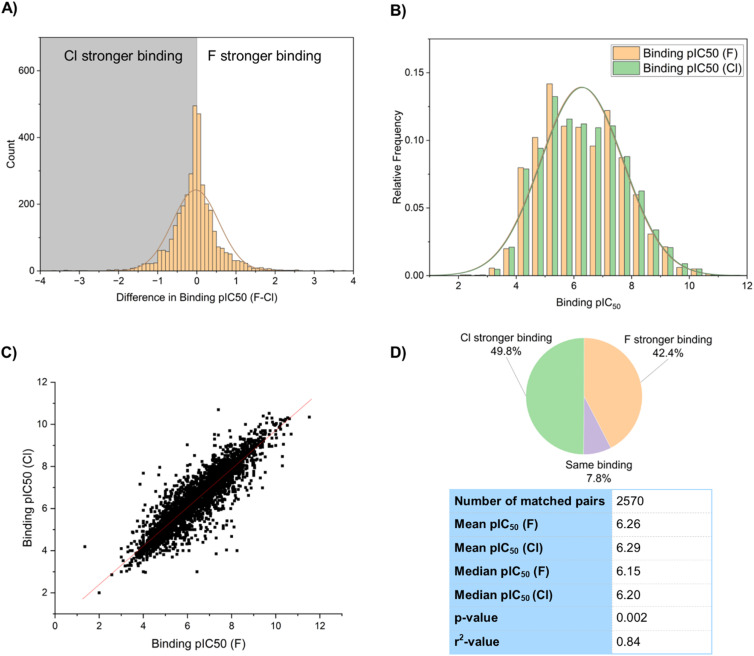
(A) Histogram showing difference in pIC_50_ values between fluorinated and chlorinated matched pairs; (B) pIC_50_ value distribution; (C) correlation between binding constants of fluorinated and chlorinated matched pairs; (D) statistical analysis of fluorinated and chlorinated matched pairs.

The binding constant of a drug molecule to its target is a particularly important parameter in predicting biological activity. In our sample of binding constants, 3611 examples of matched pairs with a fluoro to chloro replacement were selected from the database for statistical analysis. These were plotted on a histogram (Fig. 3A), and parameters describing the distribution were calculated. A disproportionate number of the selection (281) showed a change of 0 between the matched pair, and for the purpose of graphical display of the histogram, 50% of these were assumed to be a negligible decrease and 50% a negligible increase to not over-bias the 0–0.1 category.

Overall, these results showed the chlorinated compounds were slightly more active than their fluorinated counterparts with a mean difference in pIC_50_ of −0.03 (mean pIC_50_ [F] = 6.26, mean pIC_50_ [Cl] = 6.29), and a median difference in pIC_50_ of −0.05 (median pIC_50_ [F] = 6.15, median pIC_50_ [Cl] = 6.20). This corresponds to an average increase in binding constant for chlorinated compounds over fluorinated compounds of around 10–12%. Of the sample 1800 (50%) showed higher activity for the chloro compound, and 1530 (42%) showed higher activity for the fluoro compound. A *t*-test gave a *p*-value of 0.002 when comparing means of the fluorinated and chlorinated distributions, confirming that the increase in binding constant for chlorinated compounds was statistically significant. A scatter graph of the pIC_50_ values of the fluorinated and chlorinated compounds gave an *r*^2^ value of 0.84 (Fig. 3C), suggesting that binding constants of fluorinated and chlorinated matched pairs are reasonably, but not perfectly, correlated with each other. A slightly larger proportion of the sample showed stronger binding of the chlorinated matched pair (50% Cl *vs.* 42% F), which is in line with the stronger mean binding of the chlorinated compounds (Fig. 3D).

**Fig. 4:**
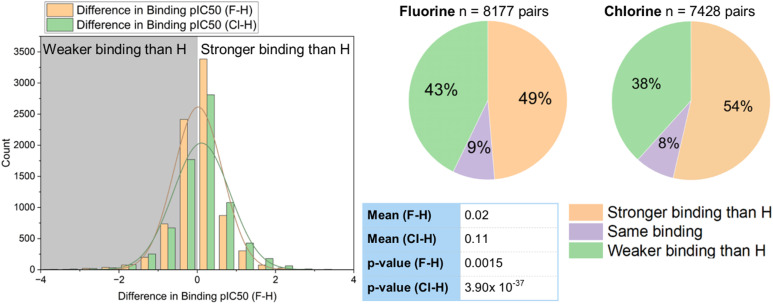
Comparison of binding of fluorinated and chlorinated compounds to hydrogen-containing matched pairs.

We also compared a series of fluorinated and chlorinated compounds to their hydrogen-containing matched pairs (Fig. 4). This showed that both halogens gave an improvement in binding over a hydrogen atom at the same position, with the improvement again being slightly larger for chlorine (mean difference F–H = 0.02; mean difference Cl–H = 0.11). Both of these improvements relative to hydrogen were shown to be statistically significant in a *t*-test (F: *p* = 0.002; Cl: *p* = 3.9 × 10^−37^). For both chlorine and fluorine the largest proportion of the sample showed stronger binding compared to a hydrogen-containing matched pair, but this proportion is greater for chlorine (54% sample for Cl *vs.* 49% F).

The key factor that leads to these slightly improved binding constants of chlorinated compounds is likely polarizability. Fluorine is very electronegative meaning its electron cloud is not easily distorted, leading to a very low polarizability (*α*_D_ = 3.74 ± 0.08), the lowest value of any element other than helium. On the other hand chlorine has a much larger polarizability (*α*_D_ = 14.6 ± 0.1).^14^ This leads to more significant induced dipole and van der Waals interactions of chlorinated molecules which could explain this slight increase in mean binding constant. A related factor that will influence the increased binding of chlorinated compounds is the increased lipophilicity of chlorine.^15^ In general, more lipophilic compounds tend to bind more strongly due to the hydrophobic effect. Additional factors that can lead to increased potency of chlorinated compounds include halogen bonding of chlorine with nucleophilic regions of a peptide backbone, and hydrogen bonding interactions of chlorine. All of these factors will be discussed in more detail with examples in the upcoming section.

**Fig. 5:**
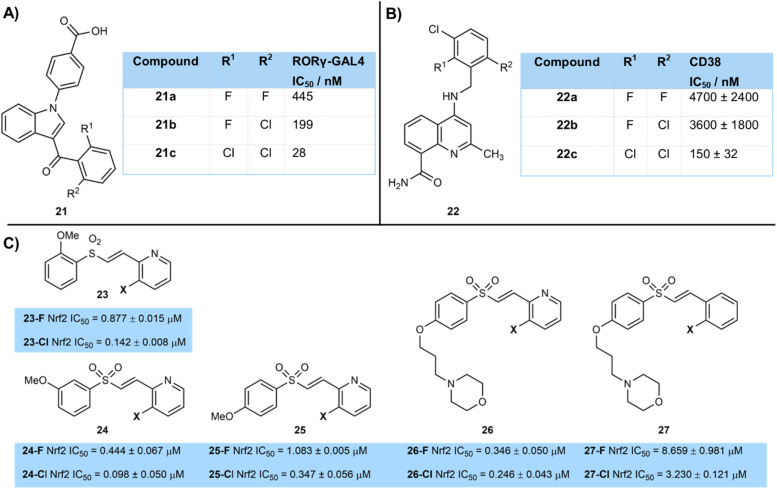
Examples of improved binding of chlorinated compounds over fluorinated matched pairs: (A) binding constants of fluorinated and chlorinated RORγt inverse agonists 21; (B) binding constants of halogenated CD38 inhibitors 22; (C) comparison of binding constants of halogenated Nrf2 inhibitors 23–27.

Shaik *et al.* reported a clear trend on chlorination of the southern phenyl ring of a series of halogenated indole derivatives as RORγt inverse agonists to treat autoimmune disorders (Fig. 5A).^16^ Dichlorinated derivative 21c (IC_50_ = 28 nM) was more active than chloro-fluoro derivative 21b (IC_50_ = 199 nM), which in turn was more active than difluorinated compound 21a (IC_50_ = 445 nM). The authors showed that bulky *ortho*-disubstitution of the aromatic ketone was important for high activity, with mono-chlorinated 21d being less active (IC_50_ = 205 nM).

Similarly, Deaton and their GSK co-workers reported 4-amino-8-quinoline carboxamides bearing a tri-halogenated aromatic ring as inhibitors of the enzyme CD38, responsible for the hydrolysis of NAD (Fig. 5B).^17^ They too showed that a tri-chloro derivative 22c (IC_50_ = 445 nM) was more active than derivatives 22b (IC_50_ = 3600 nM) and 22a (IC_50_ = 4700 nM) in which one and two chlorine atoms had been replaced by fluorine respectively.

Park and colleagues developed a series of vinyl sulfone activators of Nrf2 23–27 as a potential Parkinson's disease therapy (Fig. 5C).^18^ They showed a consistent trend of the chlorinated compounds being more active than their fluorinated analogue, regardless of the position of alkoxy substituents on the aryl ring.^19^ Activity tended to be highest when a pyridine rather than aryl ring was halogenated (*e.g.*26*vs.*27). Halogenation was only tolerated *ortho*- to the alkene substituent, with activity being lower when chlorine or fluorine were introduced to other positions.

Although the reasons for this higher activity of the chlorinated compounds were not explained in any of these three cases above, they could involve factors such as better contact in the active site of the bulkier chlorine atoms, with stronger interactions due to increased van der Waals forces. The increased lipophilicity of the chlorinated compounds will also make a contribution to their improved binding.^20^

**Fig. 6:**
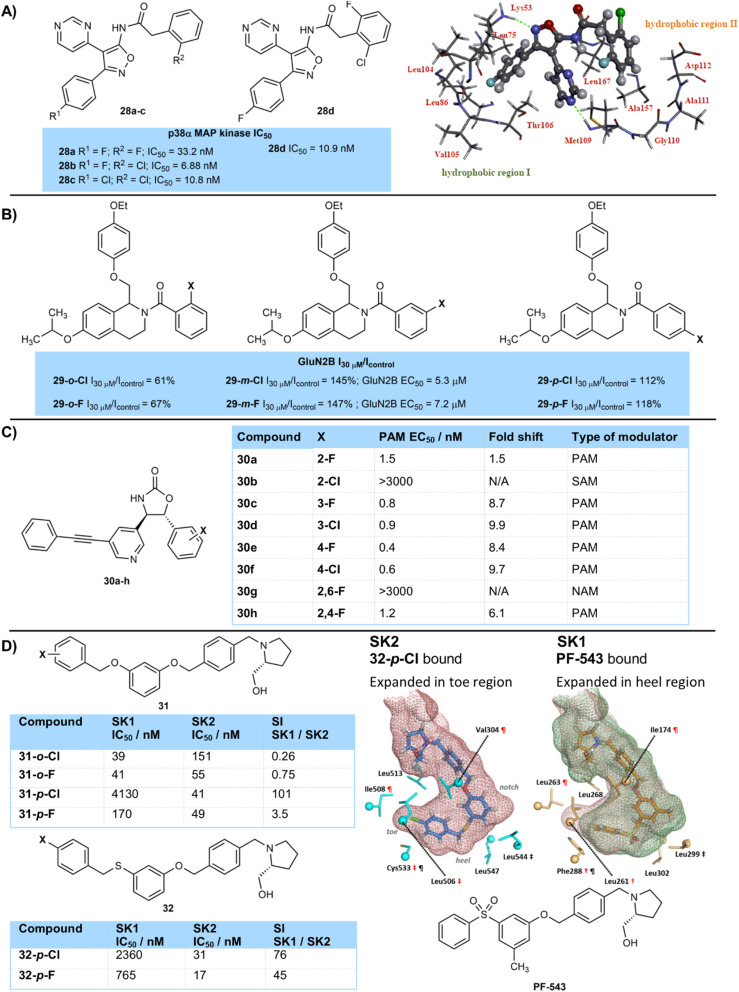
(A) Comparison of binding constants of fluorinated and chlorinated p38α-MAP kinase inhibitors, and modelled binding of compound 28d to p38α-MAP kinase. Image adapted with permission from ref. 21 (Copyright Elsevier, 2014); (B) activities of fluorinated and chlorinated GluN2B receptor modulators; (C) activities of halogenated mGluR5 allosteric modulators; (D) activities of fluorinated and chlorinated analogues of compound 31 towards sphingosine kinase enzymes SK1 and SK2, including binding to J-channel of enzymes. Image adapted with permission from ref. 26 (Copyright American Chemical Society, 2019).

However, effects of halogenation are often complex and fluorine and chlorine can often be used in combination with each other for best results. Ohta and colleagues designed inhibitors of p38α-MAP kinase as potential treatments for inflammatory bowel disease (Fig. 6A).^21^ A series of compounds were synthesised containing fluorine and chlorine atoms on two separate aromatic rings around a pyrimidine-isoxazole scaffold. Of the three matched pair compounds 28a–c most active was the mixed chloro-fluoro compound 28b (IC_50_ 6.88 nM), followed by dichlorinated derivative 28c (IC_50_ 10.8 nM), with difluorinated compound 28a the least active (IC_50_ 33.2 nM). Related compound 28d with an additional fluorine atom on the *ortho*-chlorinated ring of 28b gave a similar inhibition constant of 10.9 nM but was chosen as the candidate for further development despite being marginally less potent due to a combination of improved CYP inhibition, genotoxicity, drug efficacy, as well as good selectivity over other kinase isoforms. Docking of compound 28d into the X-ray structure of p38α-MAP kinase suggested that the 4-fluorophenyl ring sat in a hydrophobic binding pocket, whilst the other *ortho*-halogenated aryl ring sat in a second hydrophobic pocket. Induced dipole and van der Waals interactions are likely to be important molecular interactions involved in binding to these hydrophobic pockets.

The position of fluorine and chlorine atoms around an aryl ring can also often have complex effects. The team of Traynellis and Liotta have studied tetrahydroisoquinoline derivatives as potential positive allosteric modulators of the GluN2B receptor (Fig. 6B).^22^ Fluorination (29-*o*-F) or chlorination (29-*o*-Cl) at the *ortho*-position of an aryl amide led to a significant drop in current measured in a two-electrode voltage clamp recording relative to a control. However, halogenation at the *meta*-position gave significant increases in current measured in this assay, with the chlorinated analogue being more active than the fluorinated in terms of its EC_50_ value. At the *para*-position fluorination or chlorination showed little advantage over the control. In general, allosteric modulators are extremely sensitive to small structural modifications that often can lead to a large drop in activity.^23^

Huang and Degnan (Bristol-Myers-Squibb) developed mGluR5 allosteric modulators 30 based on an oxazolidinone core that highlights the sensitivity of allosteric modulators to halogenation (Fig. 6C).^24^ Whether an aryl ring was substituted with chlorine or fluorine, and the position of halogenation caused the modulators to behave as either a positive allosteric modulator (PAM), negative allosteric modulator (NAM) or silent allosteric modulator (SAM) in their effect on glutamate binding.^25^ PAM activity of fluorinated compounds (30a,c,e) was a little higher than that of chlorinated compounds (30b,d,f), and activity was higher with a substituent at the *para*-position (30e,f) compared to the *meta*- (30c,d) and especially the *ortho*-position (30a,b). Indeed, 2,6-difluorinated derivative 30g, substituted with two *ortho*-fluorine atoms, was found to be a negative allosteric modulator and glutamate antagonist, showing an antagonist IC_50_ value of 27 nM. Compound 30h fluorinated at one *ortho*- and one other ring position showed intermediate PAM activity. *Ortho*-chlorinated compound 30b was a potent silent allosteric modulator.

Pyne and colleagues have studied sphingosine kinase enzymes SK1 and SK2 with compounds 31/32, which were based on initial hit PF-543 and bind to the lipid-binding ‘J-channel’ of the enzymes (Fig. 6D).^26^ The foot of this J-channel has a different shape for the SK1 and SK2 forms and each version is better able to accommodate different halogens at different positions. SK1 is more expanded in the heel region of the foot, where an *ortho*-substituent would sit. This enzyme shows highest activity for an *ortho*-chlorine atom (31-*o*-Cl (39 nM)) and *para*-chlorine is poorly tolerated (31-*p*-Cl (4130 nM), 32-*p*-Cl (2360 nM)). SK2 is more expanded in the toe region, and best tolerates a *para*-chlorine atom (31-*p*-Cl (41 nM), 32-*p*-Cl (31 nM)) which fits well into this region. Fluorine gives a more nuanced response, binding more strongly than chlorine where the chlorine is too tight a fit (*e.g.*31-*o*-F (55 nM) *vs.*31-*o*-Cl (151 nM) with SK2, and 31-*p*-F (170 nM) *vs.*31-*p*-Cl (4130 nM) with SK1), but less strongly when the chlorine is an ideal size match and gives a better surface contact (*e.g.*31-*p*-F (49 nM) *vs.*31-*p*-Cl (41 nM) with SK2).

**Fig. 7:**
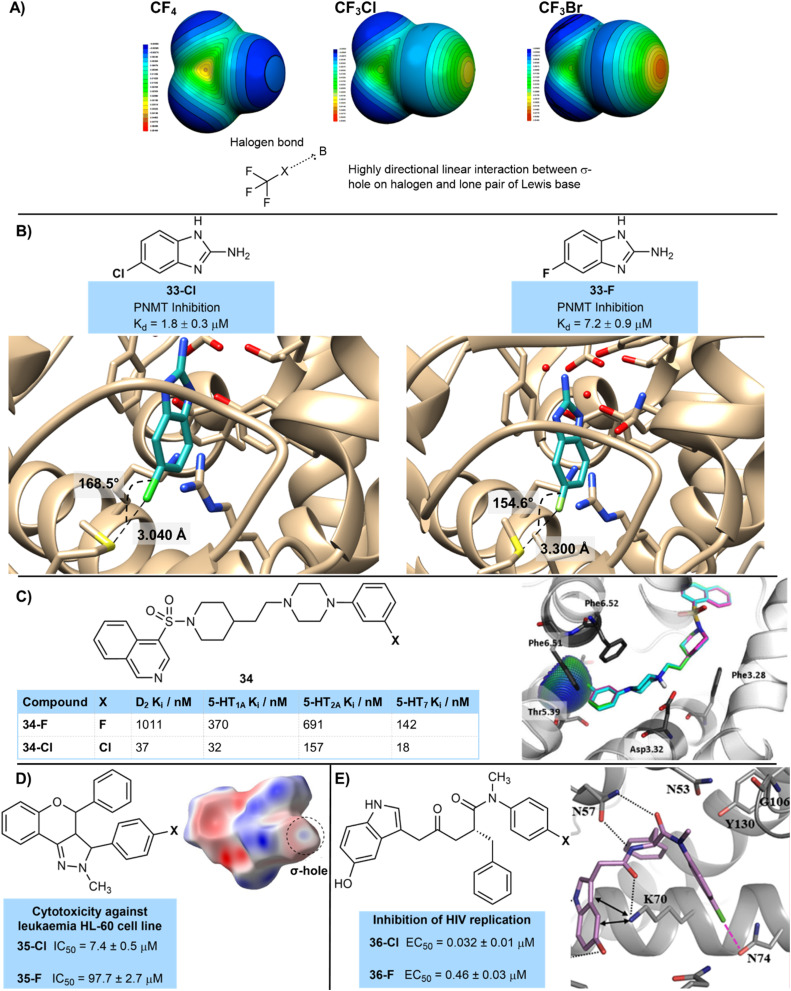
(A) Concept of halogen bonding. Image adapted with permission from ref. 27*a* (Copyright Springer Nature, 2006); (B) halogen bonding in binding of halogenated 2-aminobenzimidazoles 33 to PNMT; (C) activities of fluorinated and chlorinated 5-HT receptor antagonists showing halogen bonding interaction of 34-Cl with Thr5.39. Image reproduced with permission from ref. 29 (Copyright Elsevier, 2017); (D) activities of fluorinated and chlorinated anticancer flavonoids 35; (E) fluorinated and chlorinated HIV-1 capsid protein inhibitors, showing a key halogen bonding interaction. Image adapted with permission from ref. 32 (Copyright Elsevier, 2020).

In some cases the binding constant is significantly higher for the chlorinated compound compared to its fluorinated matched pair, which cannot be explained by the relatively small gains made by an increase in polarizability or increased surface interactions. In these cases halogen bonding is likely to be at play (Fig. 7A).^27^ Interactions of σ-holes, which are electrophilic regions of lower electron density present on heavier halogens such as chlorine, with nucleophilic regions on protein backbones such as carbonyl oxygen atoms, or sulfur, oxygen and nitrogen atoms found in side-chains are attractive and stabilizing.^27*b*^ More polarizable and less electronegative halogen atoms such as chlorine form more positive σ-holes, explaining the increased strength of halogen bonding interactions of chlorinated compounds compared to those with a fluorine atom. Of course, halogen bonding interactions of bromine and iodine are stronger and more significant than those of chlorine. These halogen bonding interactions are highly directional with a linear orientation being preferred due to the orientation of the σ-hole, and no attractive interaction being observed beyond a 40° deviation from linearity.^27*b*^

This is well-illustrated by work from Martin *et al.* who used a fragment-based approach using X-ray crystallography and ITC to identify potential PNMT inhibitors (Fig. 7B).^28^ Fluorinated and chlorinated 2-aminobenzimidazoles were identified as promising fragments, with the chlorinated analogue giving an improved dissociation constant (33-Cl*K*_d_ = 1.8 µM; 33-F*K*_d_ = 7.2 µM). The chlorinated analogue showed a halogen bond to the sulfur atom of a methionine residue (Fig. 12A), which was a more linear interaction (168.5° 33-Cl*vs.* 154.6° 33-F) and closer contact (3.040 Å 33-Cl*vs.* 3.300 Å 33-F) than that observed with fluorine. Of course, the longer nature of the C–Cl bond may also play a role in this closer interaction of the chlorinated compound, as well as a potential halogen bonding interaction.

Zajdel and co-workers have shown the importance of halogen bonding on the D_2_-and 5-HT-receptor activity of a series of halogenated azinesulfonamides (Fig. 7C).^29^ Compound 34-Cl bearing a chlorine atom at the 3-position of an aryl ring was over 10 times more active at the 5-HT_1A_ receptor than its counterpart 34-F with a corresponding fluorine atom (*K*_i_ values 34-Cl = 32 nM; 34-F = 370 nM). The authors performed molecular modelling which showed a clear halogen-bonding interaction of the chlorine atom with an oxygen of a side-chain threonine. This significantly improved binding was also reflected across several other serotoninergic receptors (5-HT_1A_, 5-HT_2A_, 5-HT_7_) as well as the dopaminergic receptor D_2_. Similar halogen bonding interactions were shown in the interaction of the chlorine atom with these other receptors.

Similarly, Kupcewicz and co-workers have suggested the importance of halogen bonding in a series of cytotoxic anti-cancer flavonoid derivatives (Fig. 7D).^30^ Against a human leukemia promyelocytic HL-60 cancer cell line compound 35-Cl containing chlorine showed an IC_50_ value of 7.4 µM, whilst fluorinated matched pair 35-F was much less active with IC_50_ = 97.7 µM. Hirshfeld surface analysis was used to correlate the importance of close contacts involving chlorine to high levels of cytotoxicity.^31^

Wang *et al.* designed inhibitors of the HIV-1 capsid protein which showed an important halogen bonding interaction that led to high activity of a chlorinated derivative (Fig. 7E).^32^36-Cl (0.032 µM) showed an over 10-fold increase in inhibition of HIV replication compared to 36-F (0.46 µM). Molecular modelling suggested a key halogen bonding interaction in the chlorinated derivative with the oxygen of an asparagine side-chain amide group N74, which was not present in the fluorinated derivative.

**Fig. 8:**
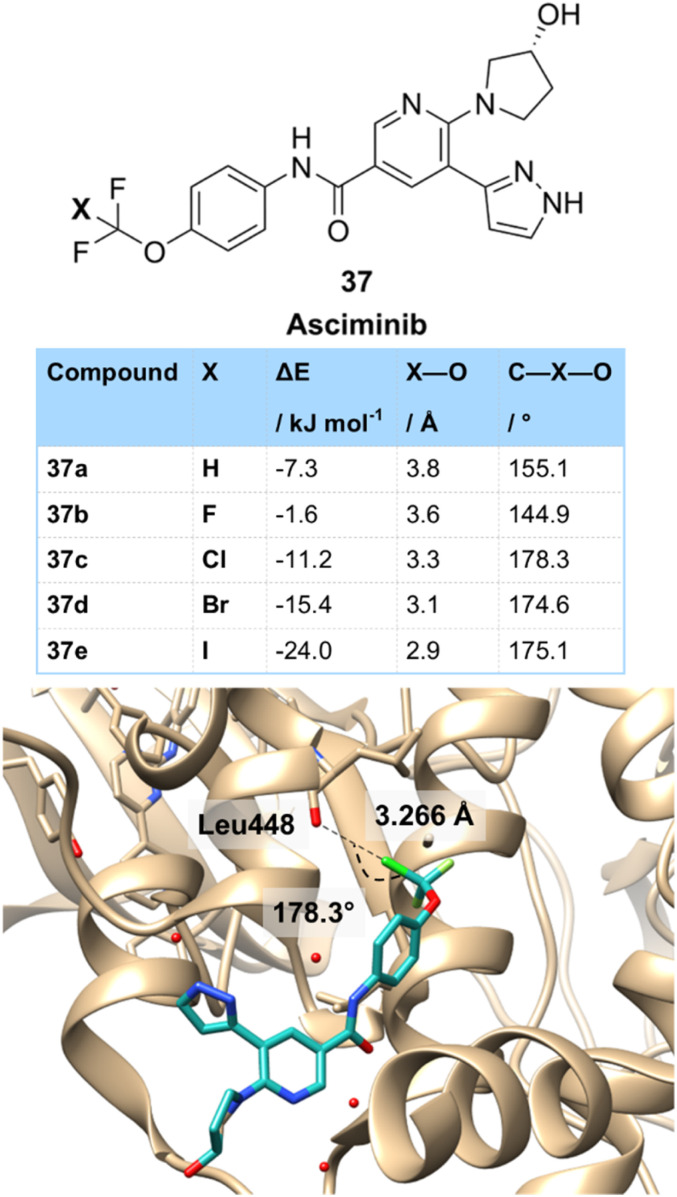
Halogen bonding in CF_2_X derivatives as demonstrated in the structure of asciminib 37c bound to ABL1 kinase.

Other more unusual motifs can participate in halogen bonding. One such motif that is seeing recent attention is the CF_2_Cl group, which has a polarized Cl atom that should form halogen bonds that are stronger than a typical chlorine. Asciminib 37c is an ABL1 kinase inhibitor used in the treatment of chronic myeloid leukaemia that contains an OCF_2_Cl group attached to an aromatic ring (Fig. 8). The Novartis team who developed asciminib were able to co-crystallize it with BCR-ABL1.^33^ Boeckler then proceeded to analyze this crystal structure in more detail, showing a key halogen bonding interaction of the chlorine with the carbonyl oxygen of Leu-448.^27*b*^ This interaction was linear (178.3°) and had a Cl–O distance of 3.266 Å. Boeckler then compared the binding of asciminib to derivatives in which the CF_2_Cl group had been replaced with other halogens using molecular dynamics at the MP2/TZVPP level of theory. This showed that the OCF_3_37b compound bound much less strongly (−1.6 kJ mol^−1^ (OCF_3_) *vs.* −10.4 kJ mol^−1^ (OCF_2_Cl)) and that the C–F–O interaction was much less linear (144.9°). The interaction with the OCF_2_H derivative 37a (−7.3 kJ mol^−1^) was stronger than the OCF_3_ due to the likely participation of the OCF_2_H group in hydrogen bonding,^34^ Finally, OCF_2_Br 37d and OCF_2_I 37e derivatives were compared which had a stronger halogen bonding interaction than OCF_2_Cl and a similar linear arrangement. However, these compounds containing the heavier halogens were much less stable than the OCF_2_Cl derivative.

Hydrogen bonding is of course another important intermolecular interaction involved in the binding of compounds. In general, the halogens participate as hydrogen bond acceptors far less than atoms such as oxygen and nitrogen, despite the high electronegativity of the halogens. This is thought to be because of the tightly-held nature of halogen lone pairs and lack of polarizability.^35^ However after significant debate,^36^ the consensus of the community is now that weak hydrogen bond interactions of the halogens is possible and that these may play a role in the binding of substrates.^37^ In general these interactions are slightly stronger with chlorine than with fluorine. For example, measurement of the intramolecular hydrogen bond strength of 2-halophenols by IR spectroscopy showed the hydrogen bond strength increased in the order F > Cl > Br > I,^38^ with doubts expressed that any hydrogen bond is present in the fluorinated compound.^39^ The balance between halogen and hydrogen bonding in these systems can be subtle and distinguishing between them can be difficult.^40^

**Fig. 9:**
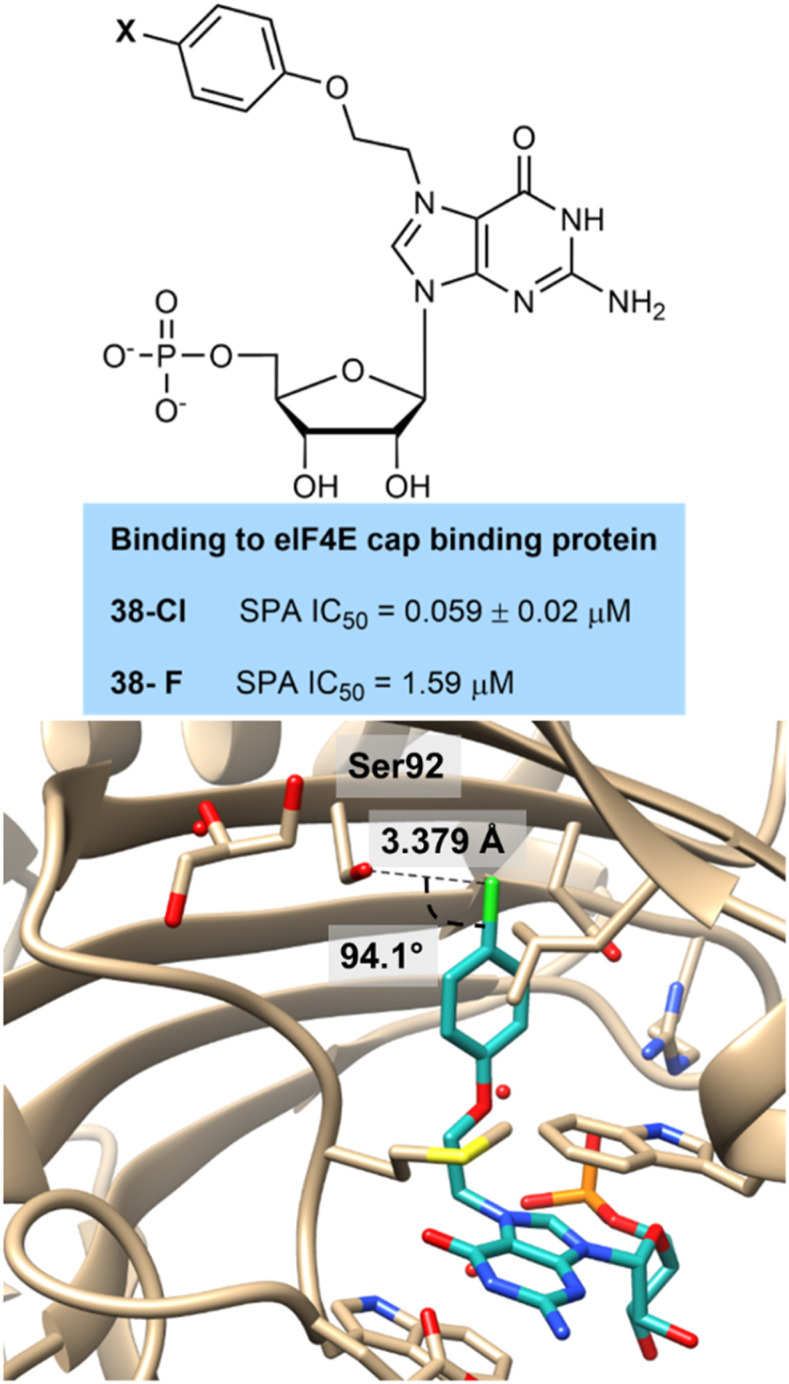
Binding of 38-Cl to active site of eIF4E showing perpendicular hydrogen bonding interaction to serine residue.

A team from Amgen led by Chen and Wang prepared guanine derived inhibitors of the eukaryotic initiation factor eIF4E involved in gene translation and protein synthesis and is overexpressed in cancer cells (Fig. 9).^41^ They showed that chlorination 38-Cl (0.059 nM) and fluorination 38-F (1.59 nM) at the para-position of an aromatic ring improved activity over a non-halogenated derivative (43 nM). This effect was repeated across other linker systems. They co-crystallised the chlorinated compound 38-Cl with the enzyme and this showed a perpendicular hydrogen bonding interaction with a hydroxyl group of a serine residue at a distance of 3.4 Å, which approaches the range expected in a hydrogen bonding interaction. Although the fluorinated derivative was not co-crystallised it would be expected to have a less strong hydrogen bonding interaction as its lone pairs are even more tightly held than the chlorinated compound.

**Fig. 10:**
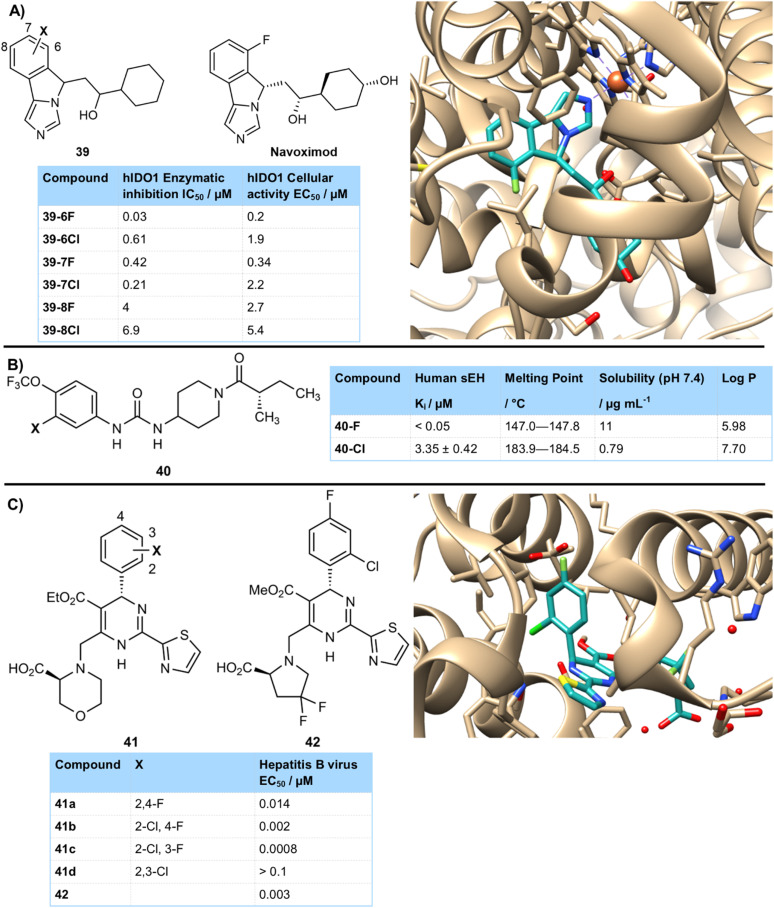
(A) Halogenated inhibitors of IDO1 showing higher activity of fluorinated derivatives and tightness of fit in binding site; (B) halogenated urea sEH inhibitors; (C) halogenated inhibitors of hepatitis B core protein illustrating tightness of binding pocket which is best able to accommodate fluorine.

Of course, there were many cases in which the fluorinated compound was significantly more active than its chlorinated counterpart. This could be due to several factors. The smaller size of a fluorine atom could make it a better fit in a binding pocket. For example, Mautino *et al.* developed imidoazo[5,1-α]isoindole inhibitors of IDO1 39 which showed a strong halogen effect (Fig. 10A).^42^ The isoindole ring sat in a tight hydrophobic binding pocket. Halogenation was poorly tolerated at C7 and especially C8 due to steric clashing with this hydrophobic cavity. However, fluorination at C6 gave a potent IDO1 inhibitor 39-6F (0.03 µM) due to a strong interaction with C-α of Gly262. The larger size of chlorine at this position was suggested to cause more steric perturbations to the enzyme at this residue and had much lower potency 39-6Cl (0.61 µM). Optimization of stereoisomers lead to navoximod which is currently in clinical trials for the treatment of advanced solid tumours. A crystal structure of navoximod bound to IDO1 showed the tightness of the active site and the challenge of accommodating atoms larger than fluorine at C6.

Hammock and colleagues prepared inhibitors of soluble epoxide hydrolase (sEH) as potential treatments for diabetic neuropathic pain (Fig. 10B).^43^ They found that derivative 40-F, containing a fluorine atom at the 3-position of an aromatic ring gave excellent activity (<0.05 µM), whilst improving solubility and lowering the melting point to assist formulation. Compound 40-Cl with a chlorine atom at the same position was much less active (3.35 µM), as well as being less soluble and having a higher lipophilicity and melting point. The authors hypothesized that the aryl substituent sits in a tight region of the binding pocket and that any substituent larger than fluorine is poorly tolerated.

A team from Roche Shanghai have reported potent inhibitors of the hepatitis B core protein, involved in assembly of the viral nucleocapsid and preventing replication (Fig. 10C).^44^ Fluorinated derivatives proved particularly effective, with a di-halogenated aryl ring required for high activity that showed a clear halogen regioisomer effect.^45^ Whilst 2,4-difluoro 41a (0.014 µM) and 2-chloro-4-fluoro 41b (0.002 µM) both showed high activity, the situation was different for 2,3-dihalo isomers. The 2-chloro-3-fluoro- isomer 41c retained high activity (0.0008 µM), but 2,3-dichloro compound 41d was inactive (>0.1 µM). Other large groups such as bromo and cyano were also not tolerated at the 3-position. This was postulated to be due to a lack of space in the binding pocket for a group or atom larger than fluorine, which was demonstrated in the crystal structure of related derivative 42 bound to the target enzyme which showed some very tight interactions.

**Fig. 11:**
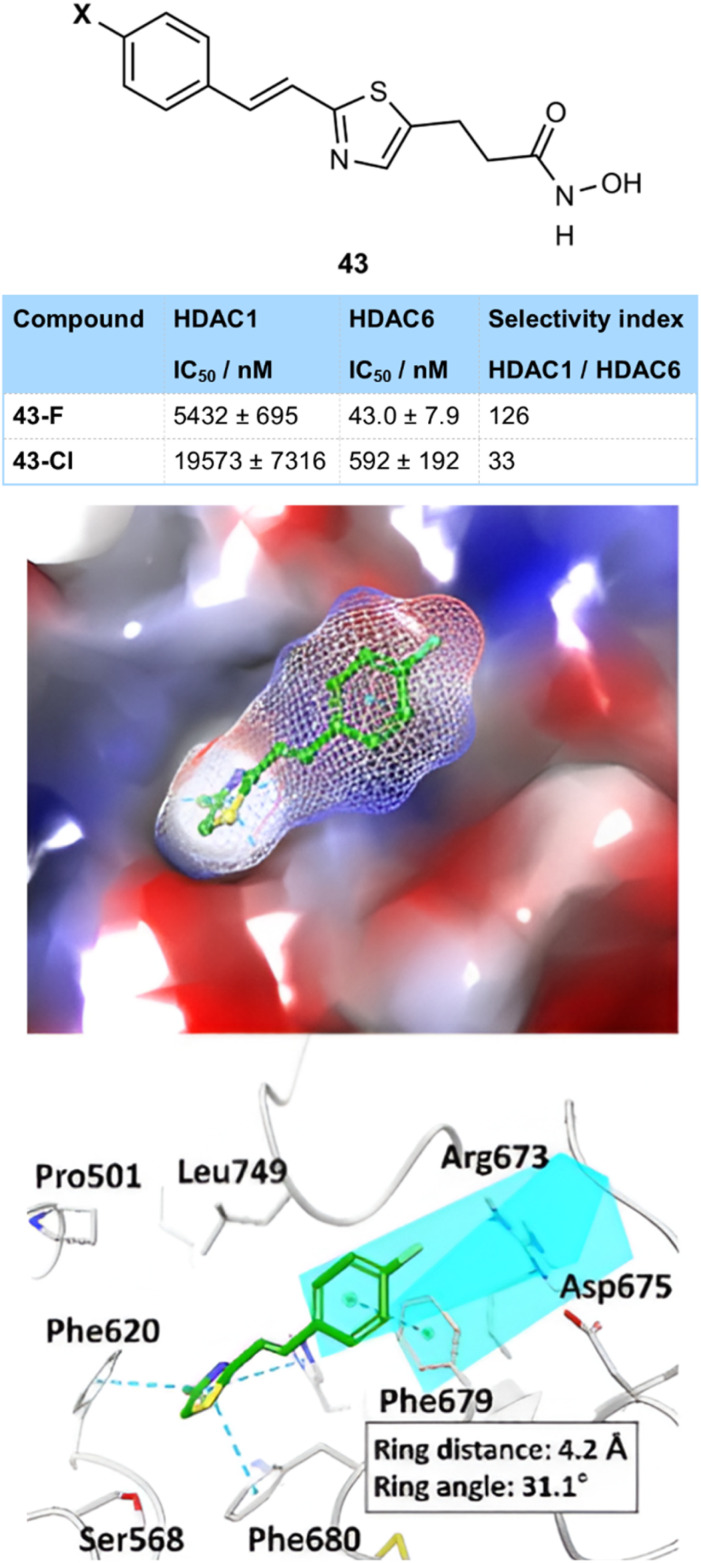
Halogenated HDAC inhibitors 43 showing an electrostatic interaction between fluorine and Arg 673, as well as π-stacking. Image adapted with permission from ref. 46 (Copyright Elsevier, 2019).

The ability of fluorine to better engage in electrostatic interactions than chlorine was demonstrated in a study of HDAC inhibitors by Jung and co-workers (Fig. 11).^46^ They found that a thiazolyl-hydroxamate substituted with a fluorine atom 43-F (43 nM) at the *para*-position of an aryl ring was much more active than the corresponding chlorinated compound 43-Cl (592 nM), and was also much more selective for HDAC-6 over other HDAC subtypes, important in reducing side effects. A docking study suggested that the *para*-fluorine substituent was involved in an electrostatic interaction with the guanidinium side-chain of Arg673 of HDAC-6, a residue which is absent from other HDAC subtypes, explaining the high selectivity observed and preference for fluorine over chlorine. A π-stacking interaction with Phe679 was also proposed, which was stronger for the more polarized fluorinated ring. In addition, the larger size of the chlorine atom was suggested to fit less well into the binding pocket.

**Fig. 12:**
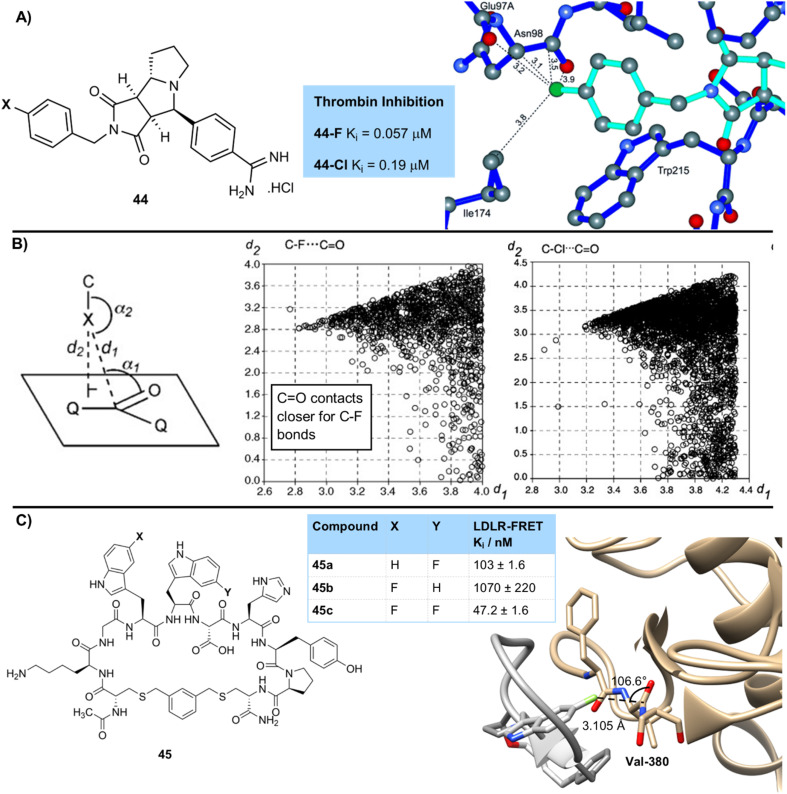
(A) Interaction of fluorinated thrombin inhibitors with peptide bonds showing close C–F to amide contacts; (B) crystallographic survey of carbon–halogen contacts with carbonyl groups. Image reproduced with permission from ref. 48 (Copyright Wiley, 2004); (C) effect of fluorination on binding of macrocyclic peptide inhibitors to PCSK9-LDLR.

A classic study by Muller and Diederich showed the importance of dipolar interactions of fluorine with amides (Fig. 12A).^47^ When studying thrombin inhibitors the team showed very high activity of a *para*-fluorobenzyl derivative 44-F (0.057 µM), which was not replicated in the *para*-chloro compound 44-Cl (0.19 µM). They showed that this high affinity for the fluorinated compound was due to a close quasiperpendicular contact (3.5 Å) with an asparagine unit in the peptide backbone. They went on to perform a search of the CSD^48^ for similar close C–F⋯C=O contacts (Fig. 12B), and showed these interactions to be quite common. This was demonstrated by a plot of distances between a fluorine atom and the carbonyl carbon against the linear distance between a fluorine atom and the plane of the carbonyl unit. This showed a narrow cone of points which indicate a preference for a fluorine atom to be at 90° to the plane of a carbonyl group at close contact distances. This analysis was repeated for chlorine, which was shown to have less preference for these interactions. The interaction distance was shown to be on average shorter for the fluorinated compounds. Similar directional dipolar interactions were shown between fluorine and nitrile and nitro substituents.^49^ Bühl and O'Hagan have shown these kinds of C–F to carbonyl interactions to be stabilising by around 1 kcal mol^−1^.^50^ No analysis has yet been performed on how much less stabilising a chlorine atom is in this situation.

An important example of these highly directional dipolar effects in binding of fluorinated compounds was demonstrated in a study by Merck of inhibitors 45 that can block the protein–protein interaction between PCSK9 and LDLR (Fig. 12C).^51^ They prepared a series of cyclic peptides containing 5-fluorotryptophan units at key positions, and compared potency to derivatives in which this had been replaced by a simple tryptophan at amino acid positions 3 and 4 in the cyclic peptide. Fluorination at position 3 gave a small improvement in potency over its non-fluorinated matched pair (47.2 nM (F, 45c) *vs.* 103 nM (H, 45a)), but the effect at position 4 was much more significant. At position 4 the fluorinated derivative was over 23-fold more potent than its non-fluorinated matched pair (47.2 nM (F, 45c) *vs.* 1070 nM (H, 45b)). This is a very significant and interesting effect considering it is due to a single atom replacement in a cyclic peptide of molecular weight >1200. The authors postulated that this was due to interactions of fluorine with backbone N–H bonds and carbonyl oxygen atoms, however a close interaction (3.105 Å) between fluorine and the carbonyl carbon of Val-380 along the Burgi-Dunitz trajectory (106.6°) was observed in the crystal structure of this compound bound to its target. This suggests that these dipolar interactions of fluorine with carbonyl groups are also important to binding in this case and demonstrate the potential magnitude of this effect. The authors have since further optimized the structure of these compounds, resulting in enlicitide decanoate, an orally available PCSK9 inhibitor that reduces cholesterol and is being developed as a treatment for atherosclerotic cardiovascular disease.^52^

**Fig. 13:**
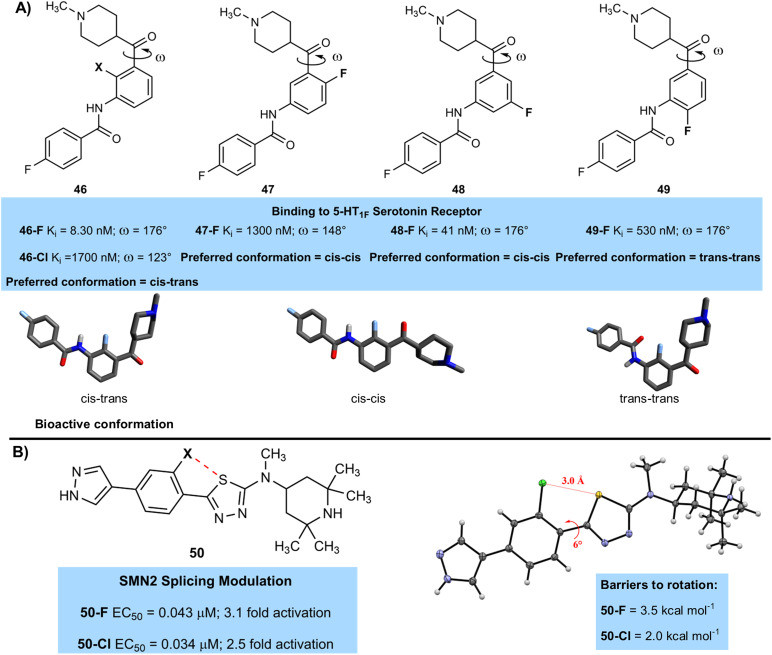
(A) Relationship between dihedral angle and activity in halogenated 5-HT_1F_ receptor agonists; (B) S–F interaction controls conformation in thia-diazole spinal muscular atrophy treatment.

Fluorination can also induce interesting conformational effects on molecules that can have significant effects on binding. A team from Lilly lead by Blanco studied some selective 5-HT_1F_ serotonin receptor agonists and showed that the fluorinated derivative 46-F (8.3 nM) had much higher affinity for this receptor than the chlorinated analogue 46-Cl (1700 nM) (Fig. 13A).^53^ The authors performed some computational studies to examine conformational effects on binding and established that the most active conformation was a *cis*–*trans* arrangement of substituents on either side of the aromatic ring, with a dihedral angle of around 180° that places the ketone co-planar with the central aryl ring. The fluorinated derivative was close to this bioactive conformation, but the chlorinated derivative showed a significant deviation, with a dihedral angle of 123° due to a steric clash between the chlorine atom and the ketone-containing side chain. Moving fluorine to other positions on the central aromatic ring (47-F (1300 nM), 48-F (41 nM), 49-F (530 nM)) led to a change to less active *cis*–*cis* and *trans*–*trans* conformations, and a subsequent drop in activity. The exact reasons for these changes in conformation were not confirmed in the study, but a combination of steric effects, intramolecular hydrogen bonding interactions or similar, and minimisation of electrostatic repulsions between electronegative atoms such as fluorine and oxygen were likely to play a role.

Other kinds of intramolecular interactions can be used to control the conformation of halogenated compounds. For example, a Novartis team lead by Dales and Hurley used 1,5-sulfur–halogen interactions to stabilise a planar conformation of a biaryl system that was essential for high activity in the treatment of spinal muscular atrophy through the development of modulators that can elevate the levels of SMN protein (Fig. 13B).^54^ The authors measured both EC_50_ values of the modulators and the level of increase of SMN protein (fold activation). Both fluorinated and chlorinated compounds 50-F (0.043 µM) and 50-Cl (0.034 µM) were similarly active, and were significantly more active than compounds with a hydrogen atom at that position 50-H (∼1 µM). X-ray crystallography confirmed the planar conformation, as well as close contacts between both chlorine and fluorine with sulfur. The Cl–S distance is shorter (3.00 Å) than the sum of the van der Waals radii of the two atoms (3.55 Å), indicating the presence of an attractive interaction. It is likely that the F–S interaction is mainly electrostatic in nature, whilst the Cl–S interaction is similar to a ‘reversed’ halogen bond, with a region of negative potential orthogonal to the C–Cl bond interacting with the C–S σ* orbital. The fluorinated compound gave stronger conformational control, with a calculated barrier to rotation of around 3.5 kcal mol^−1^, whilst the chlorinated compound had a barrier of around 2 kcal mol^−1^, suggesting the S–F interaction was stronger than the S–Cl interaction. The authors were also able to uncover from the CSD several examples of similar attractive interactions between halogen atoms and sulfur that control conformation.

**Fig. 14:**
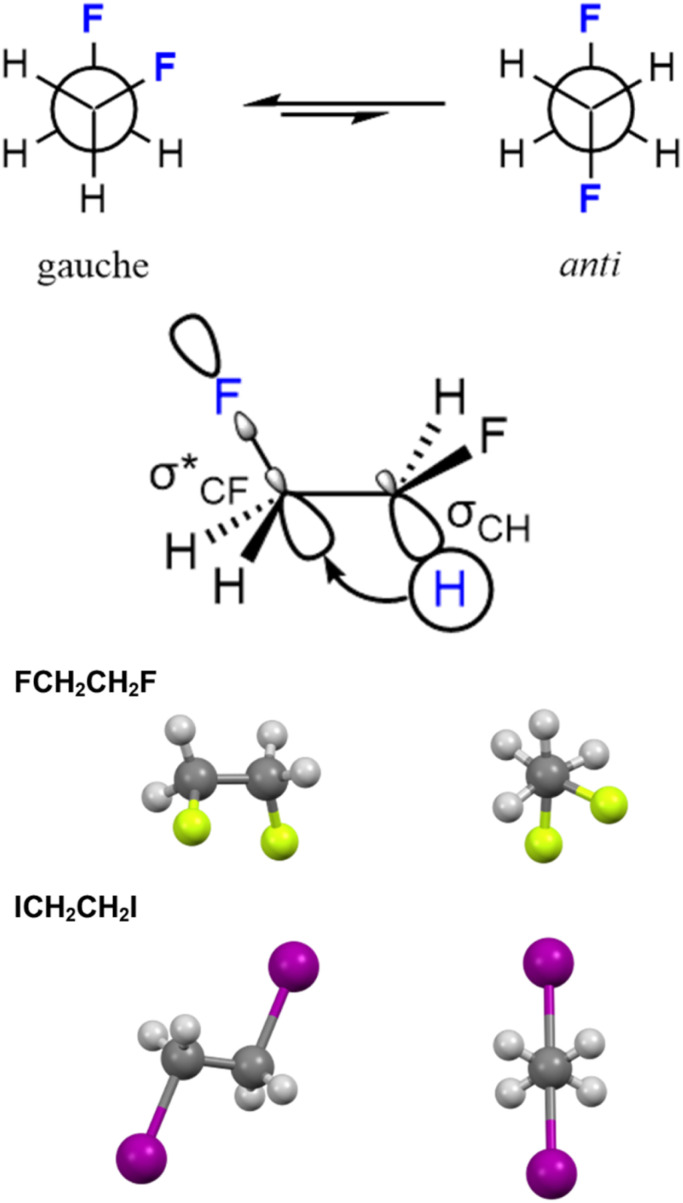
The fluorine *gauche* effect.

Another way that fluorinated compounds can exhibit different conformational preferences to chlorinated compounds is through the *gauche* effect (Fig. 14).^55^ 1,2-difluoroethane is the archetypal compound to show a *gauche* effect, and takes up a *gauche* conformation preferentially over an *anti*-conformation due to a σ_CH_ to 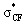 hyperconjugation interaction. 1,2-difluoroethane has a *gauche* conformation that is more stable than the *anti* in both the gas phase and solvents such as chloroform and DMSO. In addition, Lentz has demonstrated through single crystal X-ray diffraction that 1,2-difluoroethane takes up a *gauche* conformation in the solid state with a F–C–C–F torsion angle of 68°, whilst 1,2-diiodoethane takes up an *anti* conformation.^56^

Martins and Freitas have calculated the conformational preference of 1-chloro-2-fluoro-ethane and showed that, whilst the *anti*-conformation is most stable in the gas phase, it is the *gauche* conformation that is most stable in chloroform and DMSO solution, although the magnitude of this preference is much less than for 1,2-difluoroethane.^57^ This computational prediction was backed up by the magnitude of NMR coupling constants for the halo-ethanes.^58^ The larger size of a chlorine atom sterically disfavours the *gauche* conformation for 1-chloro-2-fluoro-ethane. 1,2-dichloroethane prefers an *anti*-conformation due to a dominant Pauli steric repulsion between chlorine atoms in the *gauche* conformation.^59^ The degree of importance of stereoelectronic, steric and electrostatic effects in favouring *gauche* and *anti*-conformations in 1,2-dihaloethanes is an area of much current debate.^60^

**Fig. 15:**
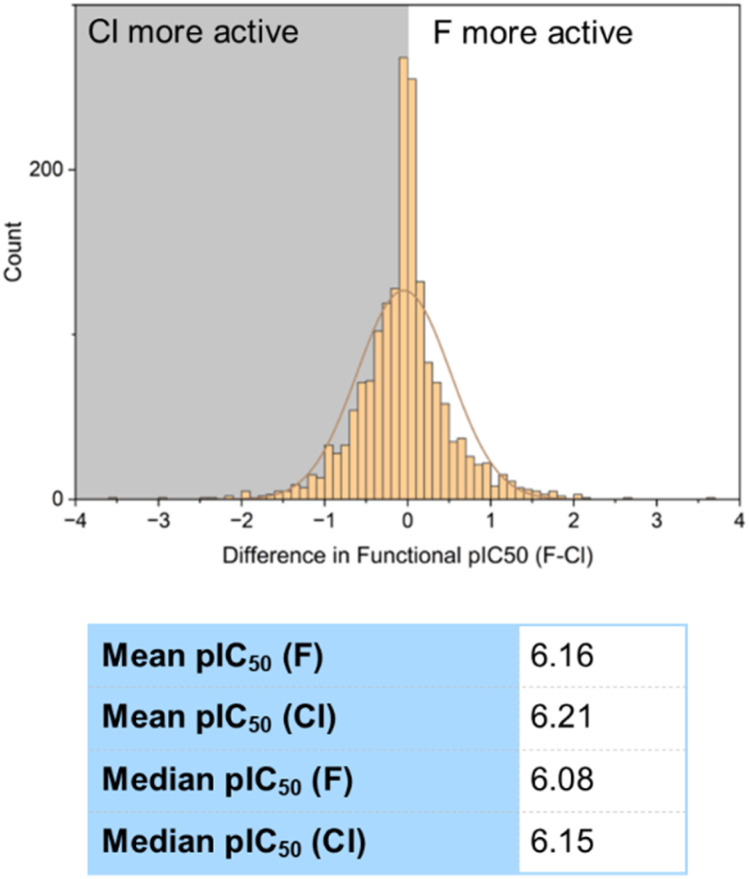
Comparison of fluorinated and chlorinated compounds in functional bioactivity assays.

Of course, an improvement in binding constant does not always lead to a corresponding increase in functional bioactivity. A similar analysis was then carried out on the functional assay data to determine whether this increase in binding strength of chlorinated compounds was carried over to observed bioactivities (Fig. 15). 1787 examples of chlorinated and fluorinated molecular matched pairs were found in our sample, of which 873 (49%) showed higher activity for the chlorinated compound and 703 (40%) higher for the fluorinated. Again, the 212 examples that showed no difference were evenly shared between a negligible decrease and negligible increase for the purpose of constructing a histogram. The mean pIC_50_ value for the chlorinated set of compounds was 6.21, and the mean for the fluorinated compounds was 6.16, showing an increase in activity of around 10% for the chlorinated compounds. This was shown to be statistically significant by a *t*-test *p*-value of 5.55 × 10^−4^. This small increase in functional bioactivity of the chlorinated compounds was supported by comparison of the median values (median pIC_50_ [F] = 6.08, median pIC_50_ [Cl] = 6.15).

Our results suggest that chlorinated compounds do on average have slightly improved binding constants compared to fluorinated compounds and this is carried over into functional bioactivity assays. However, a range of complex factors beyond simple binding affect how well a drug will perform in real situations, and which molecule would make the best drug. Toxicological, pharmacokinetic and metabolic properties will also be important, and these will be the focus of the remainder of this review.

## Toxicology

There are significant concerns about the toxicity of several classes of halogenated compounds. Polyfluoroalkyl substances (PFAS)^61^ and polychlorinated biphenyls (PCBs)^62^ are two classes of compounds that have concerns over bioaccumulation,^63^ carcinogenicity and their ability to act as endocrine disruptors, which has led to legislation restricting their use. However, it is much less clear that there are toxicology concerns over drug-like compounds bearing a low number of halogens in their structure, and many such compounds have passed clinical trials and toxicity assays with no concerns.

**Fig. 16:**
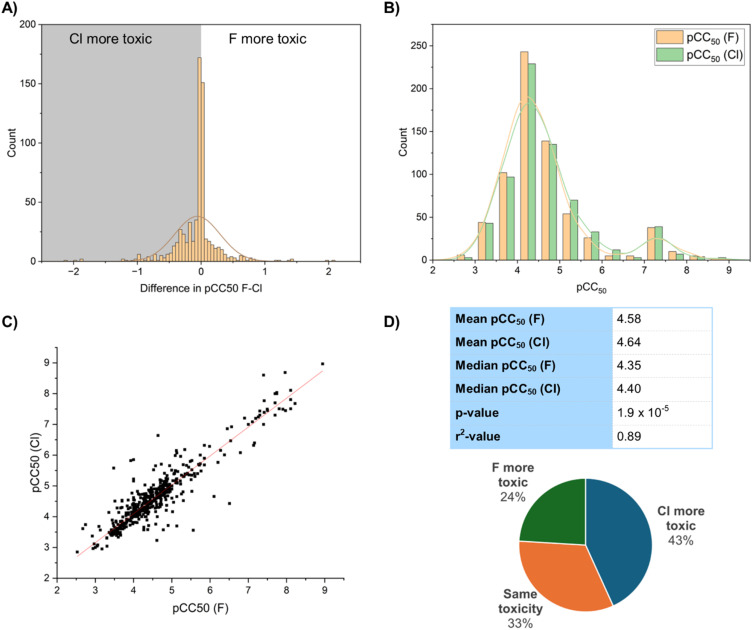
Comparison of fluorinated and chlorinated matched pairs in (A) difference in pCC_50_ values; (B) pCC_50_ value distribution; (C) correlation of pCC_50_ values; (D) statistics of pCC_50_ values.

We wanted to compare the toxicity of fluorinated and chlorinated compounds, so selected CC_50_ and LD_50_ values from the ChEMBL database and analysed these for molecular matched pairs (Fig. 16A). We found 678 examples of Cl–F matched pairs in the database of which 293 (43%) showed the chlorinated compound with higher toxicity, and 163 (24%) showed the fluorinated compound with higher toxicity (Fig. 16D). The mean pCC_50_ value of the fluorinated compounds was 4.58, whilst the corresponding mean of the chlorinated compounds was 4.64 (Fig. 16B). In terms of median values this was 4.35 for fluoro compounds and 4.40 for chloro compounds. This would suggest a 10–15% increase in toxicity of the chlorinated compounds over the fluorinated compounds. A *t*-test *p*-value of 1.9 × 10^−5^ would suggest that this increase is statistically significant. The toxicity of the chlorinated and fluorinated compounds are well-correlated with each other (*r*^2^ = 0.89) (Fig. 16C). Examination of the histogram distribution (Fig. 16A) would suggest however, that the vast majority of compounds have very little difference in toxicity when changing from fluorine to chlorine and that the results are perhaps being skewed by a small number of outliers that show a larger toxicity increase for the chlorinated compound. It is however possible that the increased lipophilicity of chlorinated compounds is leading to more non-specific off-target interactions that in turn lead to toxicity.

**Fig. 17:**
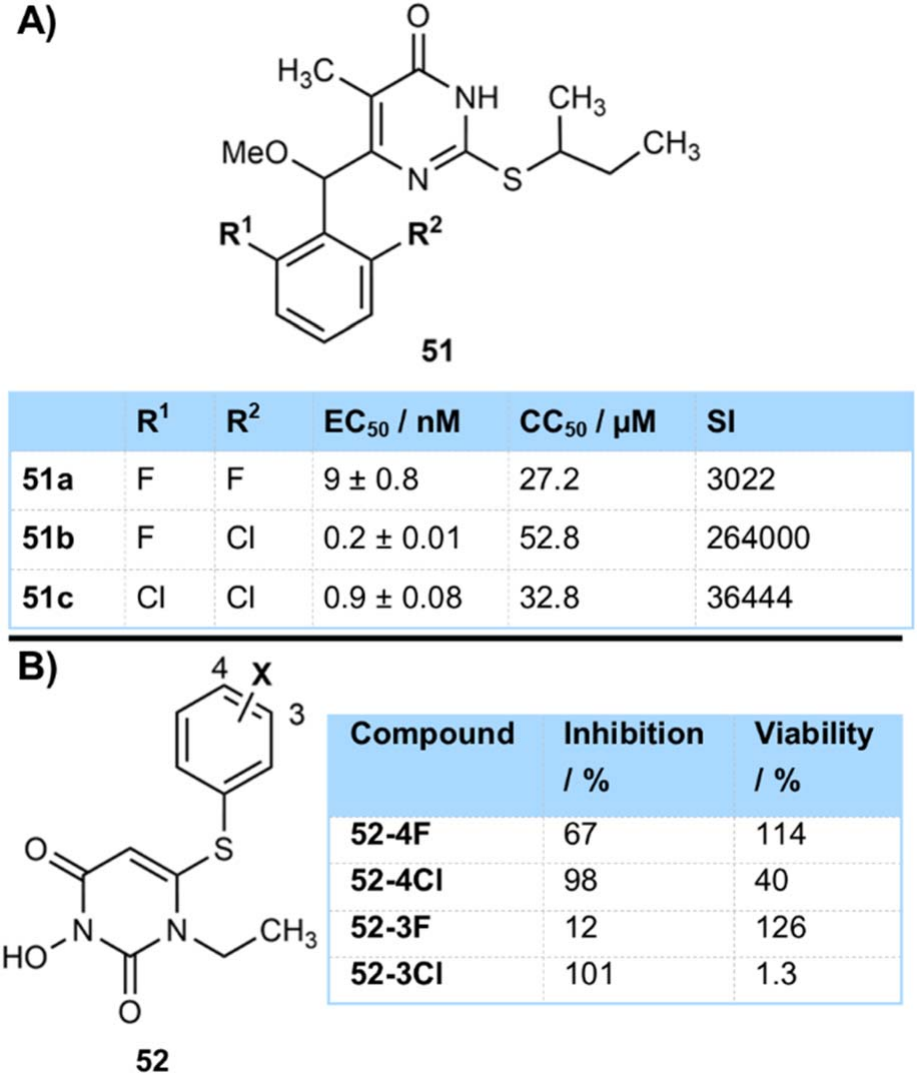
(A) Toxicology properties of halogenated HIV-1 reverse transcriptase inhibitors; (B) activity and toxicology of halogenated human cytomegalovirus inhibitors.

Esté, Mai and Rotili *et al.* compared a series of 2-chloro-6-fluoro and 2,6-dihalogenated benzenes 51 attached to pyrimidin-4(3*H*)-ones as HIV-1 reverse transcriptase inhibitors (Fig. 17A).^64^ The fluoro-chloro compound 51b (CC_50_ 52.8 µM) and dichlorinated 51c (CC_50_ 32.8 µM) were found to be perhaps marginally less cytotoxic than difluorinated compound 51a (CC_50_ 27.2 µM). The chlorinated compounds also had improved potency and therefore a better selectivity index for the best compounds. This gave compounds with wide spectrum activity against several HIV strains, including NNRTI-resistant mutants, and it was observed that enantiomers with an *R*-configuration at the α-methoxy substituent were significantly more active than those with an *S*-configuration at that position.

Wang and co-workers from Minnesota presented 6-arylthio-3-hydroxypyrimidine-2,4-dione inhibitors 52 of human cytomegalovirus which showed an interesting trend in activity and toxicity (Fig. 17B).^65^ Although the chlorinated compounds were in general better inhibitors they were also more cytotoxic than their fluorinated analogues, as demonstrated in a Hel299 cell viability assay. This effect was most pronounced on *meta*-fluorination, where the chlorinated compound 52-3Cl was extremely cytotoxic, but the safer fluorinated compound 52-3F was inactive.

## Pharmacokinetics

Halogenation has been shown to provide significant advantages in terms of the physicochemical properties of drug molecules, and likely provides bigger and more consistent improvements compared to those observed in binding. In this section we will compare the properties of fluorinated and chlorinated compounds in terms of lipophilicity and solubility, as well as in various bioavailability parameters.

**Fig. 18:**
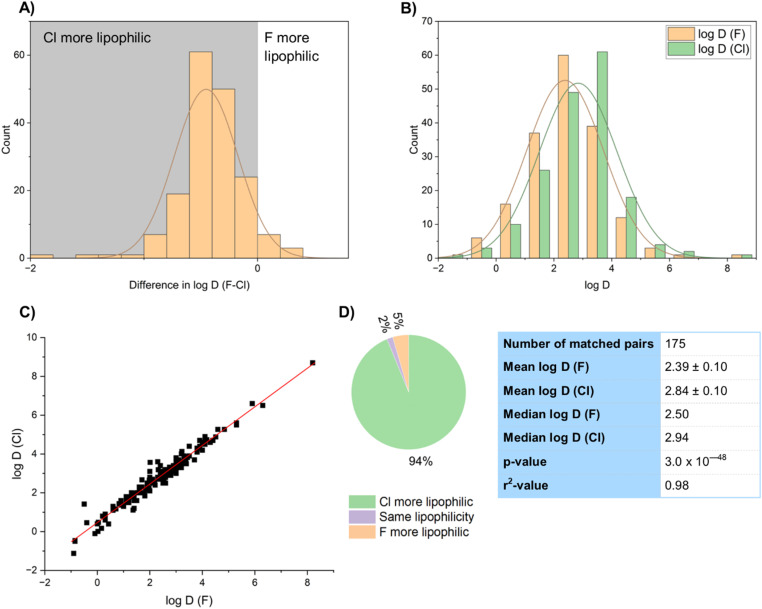
(A) Comparison of log *D* differences between fluorinated and chlorinated matched pairs; (B) comparison of log *D* differences between fluorinated and chlorinated matched pairs; (C) correlation of log *D* values of fluorinated and chlorinated matched pairs; (D) statistics of log *D* values.

Compounds containing chlorine are clearly more lipophilic than those containing fluorine (Fig. 18A). Comparing log *D* values of 177 matched pair examples showed the mean of the chlorinated compounds (2.84) to be 0.45 units higher than that of the fluorinated compounds (2.39) (Fig. 18B). The certainty of the significance of this difference in mean was confirmed by a *t*-test *p*-value of 3.03 × 10^−48^. The substituent π-values for F and Cl in aromatic systems have been reported to be 0.14 and 0.71 respectively.^66^ The difference between these values is 0.57, which is in good agreement with the average value (0.45) obtained in this study. Values of log *D* of the fluorinated and chlorinated matched pairs were very well correlated with each other, with an *r*^2^-value of 0.98 (Fig. 18C). In addition, 94% of the sample showed a higher log *D* for the chlorinated matched pair, confirming the higher lipophilicity of chlorine (Fig. 18D).

**Fig. 19:**
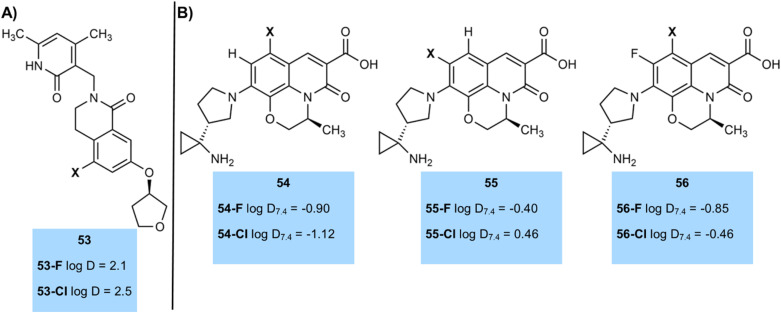
(A) log *D* of halogenated EZH2 inhibitors; (B) log *D* of halogenated fluoroquinolone antibacterials.

Again, we wanted to demonstrate these patterns with real examples. For example a Pfizer team prepared a series of dihydroisoquinoline-1(*2H*)-ones as EZH2 inhibitors (Fig. 19A).^67^ The fluorinated derivative 53-F (log *D* = 2.1), with its fluorine atom relatively distant from other functionality had a log *D* value approximately 0.4 log *D* units lower than the chlorinated derivative 54-Cl (log *D* = 2.5), close to the average value.

Whilst the distribution of log *D* difference values were certainly clustered between the fluorinated compounds being between 0.2 and 0.6 log *D* units lower than their chlorinated counterparts (with 70% of the dataset falling within this range), there were also certainly some outliers to this pattern.^68^ There has been some discussion of the kinds of fluorinated motifs that lead to these outlier results, although more work is certainly needed to understand them better. Alkyl fluorination often lowers log *P*, particularly if there are nearby electronegative vicinal or proximal oxygen or halogen atoms.^69^ Whilst aromatic fluorination normally increases log *D* relative to a hydrogen at the same position, *ortho*-substituted alcohols, ethers and carbonyl compounds can show a lowered log *D*. It is thought that this is due to an increase in the overall polarity of the molecule. On the other hand, certain compounds containing nitrogen have been shown to have a much larger increase in log *D* on H/F exchange than is typical.^69*a*^ This is thought to be because of reduced basicity due to fluorination. To date there have been fewer efforts examining similar effects on chlorination.

The complexities of comparing log *D* values were exemplified in a Novartis study of some fluoroquinolone antibacterial agents (Fig. 19B), where the number and position of halogen atoms on the fluoroquinolone ring had significant effects on log *D*.^70^ Halogenation at C6 gave a matched pair 54 with the chlorinated analogue actually having a lower log *D* than the fluorinated analogue by 0.22 log *D* units. Conversely, halogenation at C5 (compound 55) gave the expected result of the fluorinated compound having a lower log *D* value, but the difference between the matched pair was 0.86 log *D* units, which was one of the largest we found in our study. Compound 56, with fluorination at C6 and either chlorination or fluorination at C5 gave a more typical result of the difluoro compound 56-F having a log *D* value 0.39 log *D* units lower than its chloro-fluoro analogue 56-Cl. More work needs to take place to understand some of these outlier results in log *D* values.

**Fig. 20:**
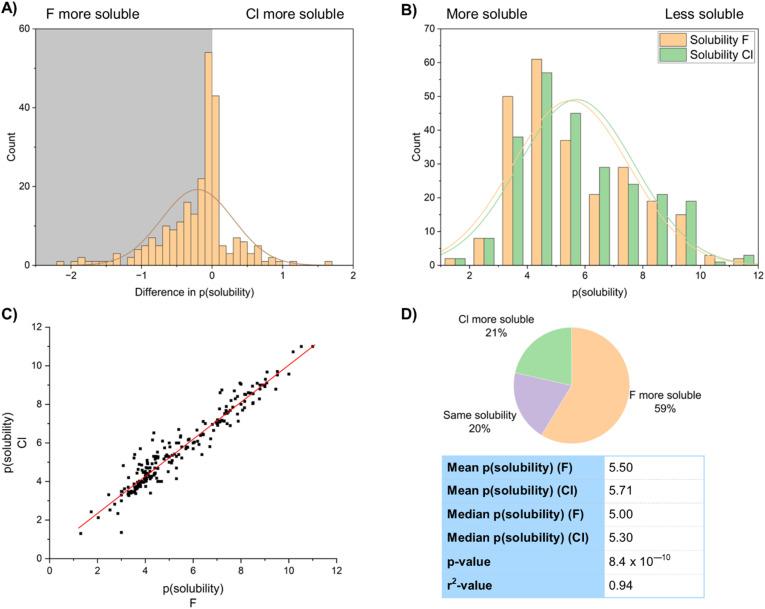
(A) Comparison of solubility differences between fluorinated and chlorinated matched pairs; (B) comparison of solubility values between fluorinated and chlorinated matched pairs; (C) correlation of solubility values of fluorinated and chlorinated matched pairs; (D) statistics of solubility values.

Similarly clear effects are shown in the solubility of fluorinated compounds (Fig. 20). Fluorinated compounds are more soluble than chlorinated compounds. The mean value of solubility of the fluorinated group of 247 compounds was 3.17 µM, whilst the mean solubility of the chlorinated matched pairs was 1.96 µM, an increase in solubility of around 60% (Fig. 20A and B). Of the dataset 59% of the compounds showed higher solubility for the fluorinated compound, 21% higher for the chlorinated compound and 20% showed no difference (Fig. 20D). This is shown in logarithmic form in the histogram. This result was backed up by a highly statistically significant *p*-value of 8.4 × 10^−10^. The solubility of chlorinated and fluorinated compounds are very well correlated with an *r*^2^ value of 0.94 (Fig. 20C).

**Fig. 21:**
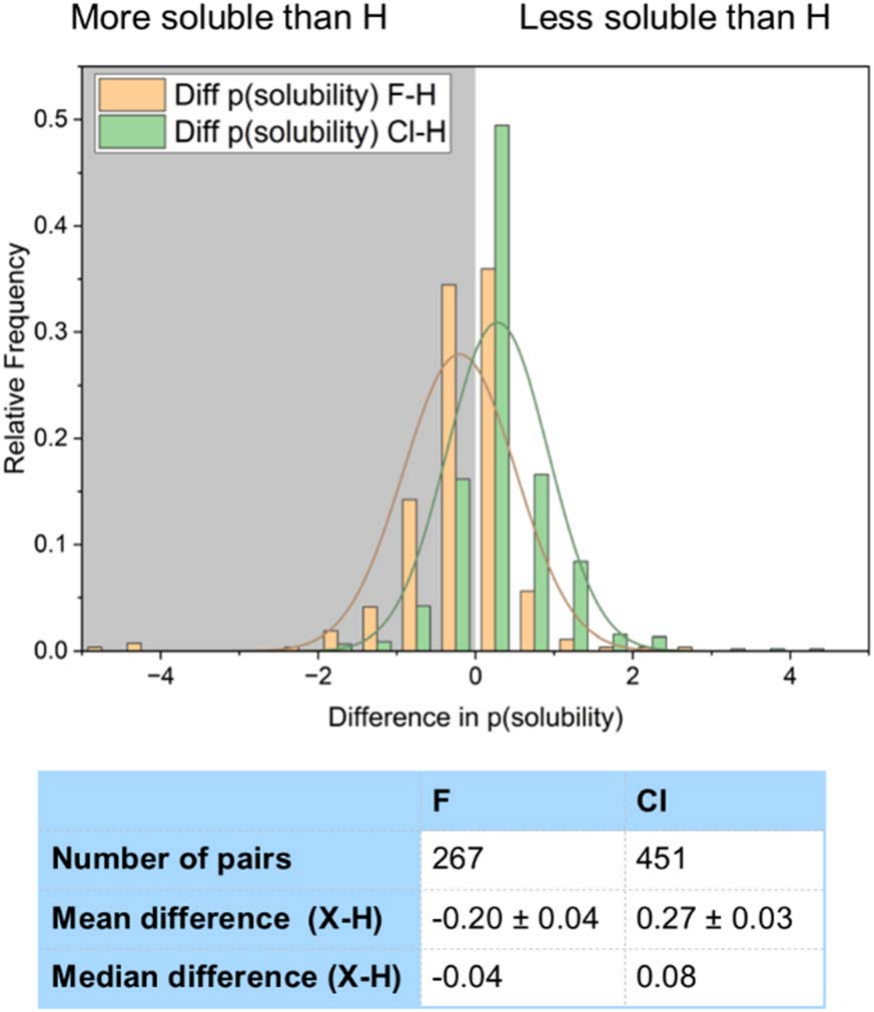
Difference in log(solubility) between F–H/Cl–H matched pairs.

Fig. 21 compares the solubility of both fluorinated and chlorinated compounds to their corresponding hydrogen-containing matched pairs. A sample of 267 fluorinated compounds and 451 chlorinated compounds were analysed. This showed that whilst fluorination on average improved solubility, chlorination decreased solubility relative to a hydrogen. 57% of the fluorinated compounds showed improved solubility relative to a hydrogen, compared to only 22% of the chlorinated compounds. Our data suggested a mean increase in solubility of the fluorinated compounds of 0.20 log units relative to a H-containing matched pair, compared to a mean decrease in solubility of 0.28 log units for the chlorinated compounds. There are therefore significant advantages in fluorination over chlorination in terms of solubility.

**Fig. 22:**
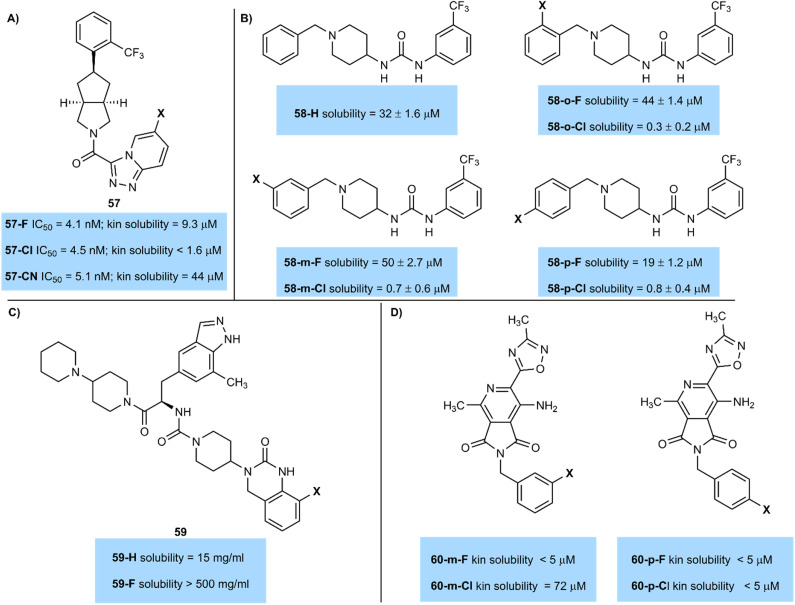
(A) Solubility of halogenated RBP4 antagonists; (B) solubility of halogenated piperidinyl ureas; (C) solubility of fluorinated CGRP antagonists; (D) solubility of halogenated antimycobacterials.

In a study of antagonists of retinol-binding protein-4 (RBP4), fluorinated and chlorinated derivatives both gave similar activity (IC_50_57-F = 4.1 nM; IC_50_57-Cl = 4.5 nM).^71^ However, the fluorinated compound presented significant advantages in terms of its kinetic aqueous solubility measured in a pH 7.4 PBS buffer (kinetic solubility 57-F = 9.3 µM; 57-Cl < 1.6 µM) (Fig. 22A). Study of both these compounds was ultimately discontinued in favour of nitrile-substituted 57-CN, which exhibited even greater solubility (44 µM) and improved metabolic properties compared to either halogenated derivative.

Similarly, Guy and co-workers prepared piperidinyl ureas 58 that target the DCN1 enzyme that is associated with squamous cell carcinoma. These compounds showed significant advantages in solubility on fluorination (Fig. 22B).^72^ At either *ortho*-, *meta*-, or *para*-positions of an otherwise unsubstituted aromatic ring fluorinated compounds were over an order of magnitude more soluble than their chlorinated matched pairs. Indeed, the *ortho*- and *meta*-fluorinated compounds 32-*o*-F (44 µM), 32-*m*-F (50 µM) were more soluble than an unsubstituted aromatic ring 58-H (32 µM), highlighting the advantages of ring fluorination in improving solubility.

Enhancements in solubility on fluorination are sometimes significant, as demonstrated by Degnan and team (BMS) (Fig. 22C).^73^ The team desired a very high aqueous solubility of an antagonist of the human CGRP receptor to support intranasal delivery as a potential migraine treatment. Solubility of 59 was enhanced from 15 mg mL^−1^ (59-H) to >500 mg mL^−1^ (59-F) by the introduction of a fluorine atom on an indazole ring. The authors hypothesized that fluorine polarized a nearby urea NH bond, improving solubility in water by making the urea a better hydrogen bond donor. Although an analogous chlorinated example was not reported, this demonstrates how fluorine can be used to give large increases in solubility in some cases.

As ever there are outliers to this trend. For example, Chibale led a study into antimycobacterials which produced pyrrolo-[3,4-*c*]pyridine-1,3(2*H*)-dione derivatives 60 (Fig. 22D).^74^ Compound 60-*m*-Cl, with a chlorine atom at the *meta*-position of an aromatic ring had significantly higher solubility (72 µM) than its fluorinated counterpart 60-*m*-F (<5 µM). However, when the compounds were halogenated at the *para*-position both fluorinated and chlorinated compounds 60-*p*-F and 60-*p*-Cl had similarly low solubility. More work is clearly needed to understand subtle solubility effects of halogenated compounds.

**Fig. 23:**
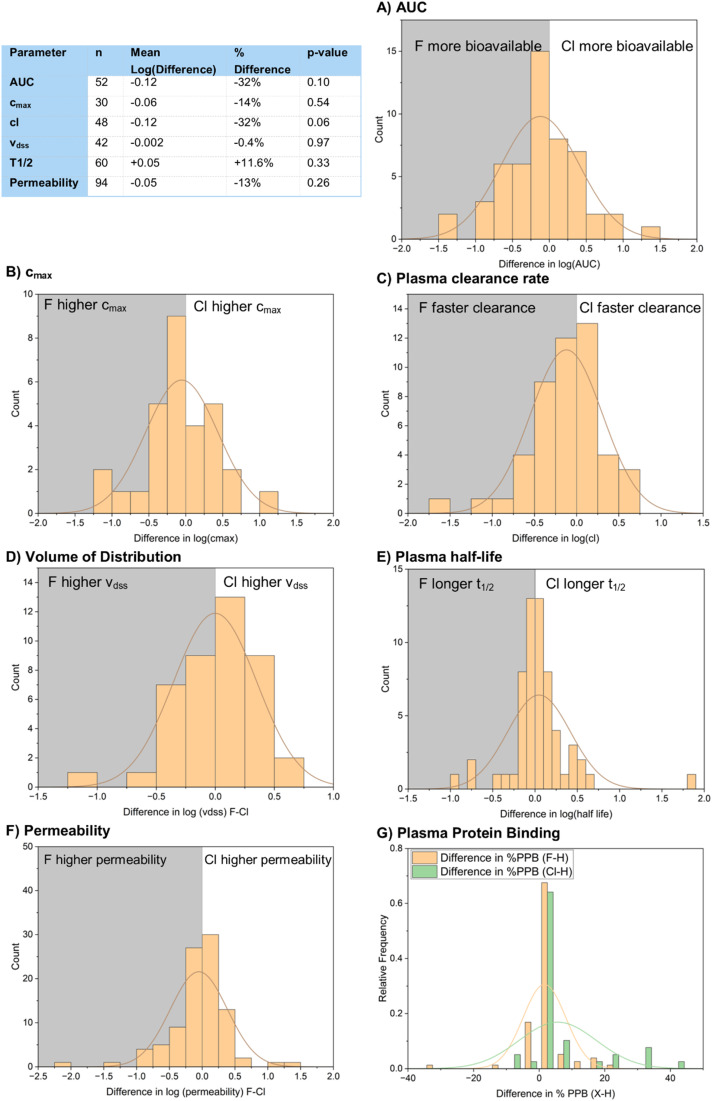
Comparison of pharmacokinetic measurements of bioavailability between fluorinated and chlorinated matched pairs (A) half-life; (B) AUC; (C) *c*_max_; (D) *v*_dss_; (E) cl; (F) permeability; (G) plasma protein binding.

Measures of bioavailability generally showed very similar performance of both chlorinated and fluorinated compounds (Fig. 23). *c*_max_, *v*_dss_ and permeability all showed no statistically significant difference in their means for the fluorinated and chlorinated compounds. AUC and plasma clearance rate gave results that were closer to showing a statistically significant difference. AUC (Fig. 23A) gave a mean pAUC value of 6.81 for the fluorinated compounds and 6.93 for the chlorinated compounds. This represents a 32% increase in bioavailability of the fluorinated compounds with a *p*-value of 0.10. Plasma clearance rate (Fig. 23C) gave a mean pCl value of 7.87 for fluorinated compounds and 7.99 for chlorinated compounds, representing an increase in clearance rate of 32% for the chlorinated compounds with a *p*-value of 0.06.

**Fig. 24:**
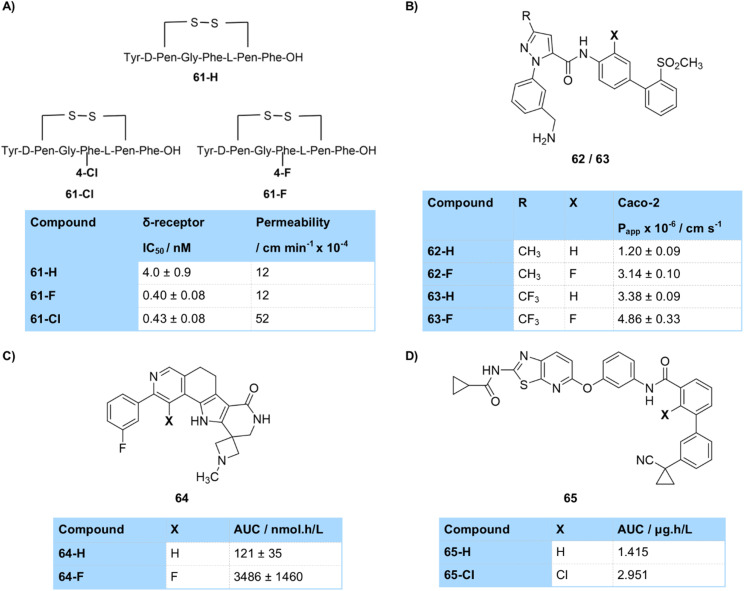
(A) Blood–brain barrier permeability of halogenated enkephalin peptides; (B) permeability of ortho-amide substituted inhibitors of coagulation factor Xa; (C) AUC of pyrrole-containing MK2-inhibitors; (D) AUC of amide-containing VEGFR2 inhibitors.

Permeability across the blood–brain barrier is one area where changing halogen atom has been shown to make a difference. Hruby and Davis *et al.* prepared analogues of the peptide analgesic enkephalin bearing different halogen atoms at the 4-position of a phenylalanine residue (Fig. 24A).^75^ The fluorinated and chlorinated matched pairs both displayed similarly high binding affinity for the rat δ-opioid receptor with high selectivity over other opioid receptors. They showed that, while blood–brain barrier permeability was not correlated to log *D* of the peptides, the chlorinated analogue 61-Cl displayed significantly better blood–brain barrier permeability than the fluorinated compound 61-F, which had very similar permeability to the unsubstituted parent compound 61-H, and that all analogues showed superb *in vitro* stability. They suggested that a variety of factors affected permeability across the blood–brain barrier, including hydrogen bonding, electrostatics, steric bulk and active transport mechanisms.

One area where halogenation has proven particularly important in improving bioavailability is in the case of amide-based drugs, where the high polarity of amides and their ability to act as a hydrogen bond donor to water often leads to poor permeability and bioavailability. Installation of a fluorine or chlorine atom *ortho* to an amide substituent has proven a popular strategy to improve bioavailability. This is due to electrostatic or intramolecular hydrogen-bonding-like interactions between the amide and the *ortho*-halogen atom that lead to desolvation of the amide and improved bioavailability. This has been demonstrated in the study of inhibitors of coagulation factor Xa by Pinto *et al.*, who showed permeability as measured in the Caco-2 assay could be improved on *ortho*-fluorination in two series of amides 62/63 (*e.g.* Caco-2 P_app_62-H = 1.2 × 10^−6^ cm s^−1^; 62-F = 3.1 × 10^−6^ cm s^−1^) (Fig. 24B).^76^ Similarly, *ortho*-chlorination of amide-containing VEGFR2 inhibitors by Ishikawa led to a large improvement in bioavailability of 65 as measured by AUC in mouse PK (AUC 65-H = 1.415 µg h L^−1^; 65-Cl = 2.951 µg h L^−1^) (Fig. 24D).^77^ Other groups which can engage in similar intramolecular hydrogen bonding interactions can also be used to improve bioavailability through desolvation, as demonstrated by Velcicky who prepared pyrrole-containing compounds which had a close interaction of a fluorine atom with the pyrrole NH (Fig. 24C).^78^ These showed significantly improved bioavailability for the fluorine-containing 64-F compared to its non-halogenated matched pair (AUC 64-H = 121 nmol h L^−1^; 64-F = 3486 nmol h L^−1^). The nature of some of these interactions of amides with *ortho*-fluorine atoms has been studied by crystallography, as well as by NMR and DFT calculations, which have suggested weak hydrogen-bonding interactions to be present.^36,79^

We also wanted to analyze plasma protein binding (Fig. 23G). Unfortunately, our dataset only provided 17 examples of direct F to Cl matched pairs, making it difficult to get significant results. Instead, we chose to indirectly compare the two halogens *via* their difference in % plasma protein binding to their hydrogen-containing counterparts. 77 examples of F–H matched pairs and 39 examples of Cl–H matched pairs were found in the dataset. The raw means of each dataset were not comparable as different compounds featured in both datasets, but we felt that it would be appropriate to compare the mean increases in plasma protein binding of the halogenated compounds to their hydrogen-containing matched pairs. The fluorinated compounds showed a mean increase in binding of 1.6%, whilst the chlorinated compounds showed a mean increase in binding of 5.6%. This result was shown to be statistically significant by a *t*-test *p*-value of 0.05, suggesting that plasma protein binding is a little more problematic for chlorinated compounds compared to fluorinated ones.

Overall, this analysis has shown fluorine to have significant advantages over chlorine in terms of improved solubility and lower log *D*, but that compounds containing either halogen appear to behave very similarly in terms of bioavailability.

For all of these measures of bioavailability a lack of direct matched pair data in the literature is making it more difficult to draw firm conclusions. It is hoped that as more high-throughput measures of bioavailability become available, significantly more matched-pair data of physicochemical and pharmacokinetic properties will be obtainable allowing for a more thorough analysis.^80^

## Metabolism

Fluorine and chlorine have both been chosen to improve the metabolic performance of drug targets. The strength of the carbon–halogen bond means that the introduction of fluorine and chlorine can be ideal to block metabolic processes. Despite this there are situations in which the halogens can be metabolically active.^81^

We wanted to use our dataset to see if either of the two halogens presented a significant advantage over the other in blocking metabolic processes at vulnerable sites. We compared % metabolic stability, metabolic clearance rate and metabolic half-life of our chlorinated and fluorinated compounds (Fig. 23).

**Fig. 25:**
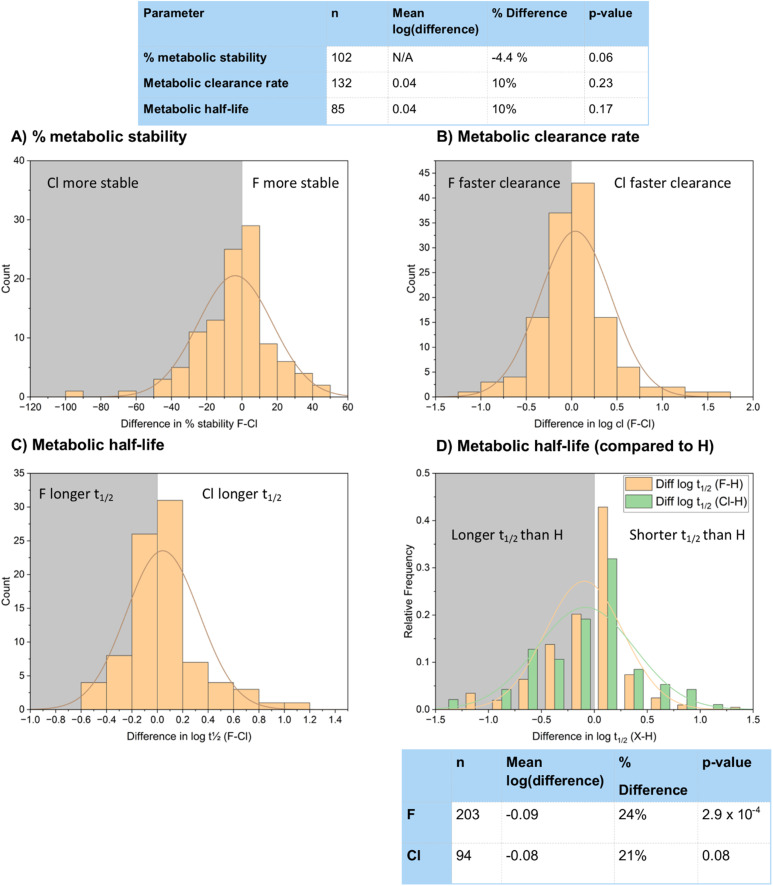
Comparison of metabolic properties of fluorinated and chlorinated matched pairs; (A) % metabolic stability; (B) metabolic clearance rate; (C) metabolic half-life; (D) metabolic half-life (compared to H).

For % metabolic stability (Fig. 25A) 109 matched pair examples were found in the dataset. This gave a mean stability of 60.0% for the fluorinated compounds and 63.8% for the chlorinated compounds. This increase in metabolic stability of the chlorinated compounds was found to be close to being statistically significant with a *t*-test *p*-value of 0.06.

The mean log Cl values (Fig. 25B) were 0.041 (10%) higher for chlorinated compounds, but a *t*-test *p*-value of 0.23 cast doubt on the statistical significance of this result. On the other hand, mean log *t*_1/2_ values (Fig. 25C) were 0.043 (11%) higher for chlorinated compounds than fluorinated compounds, although again with a non-significant *p*-value of 0.17.

We also wanted to highlight the advantages of halogenated compounds over non-halogenated compounds in blocking metabolism, so performed a matched pair analysis of both fluorinated and chlorinated compounds with their hydrogen-containing counterparts (Fig. 25D). Of the 203 fluorinated and 94 chlorinated compounds sampled, 63% of the fluorinated compounds showed identical or improved metabolic half-lives relative to a hydrogen-containing matched pair, compared to 68% for the chlorinated compounds. This corresponded to a mean increase in log(*t*_1/2_) of 0.10 log units for the fluorinated compounds with a statistically significant *p*-value of 2.88 × 10^−4^. For the chlorinated compounds the increase was similar at 0.08 log units, although the larger *p*-value of 0.08 may have been a consequence of the smaller sample size.

On balance this suggests very similar behaviour of fluorinated and chlorinated compounds in metabolic processes, and both are very good options to block undesired metabolism when this becomes an issue. The halogens are particularly effective at blocking metabolism on aromatic rings, where their high electronegativity and strong C–X bonds lead to slowed metabolic clearance. However in certain aliphatic systems, particularly in the presence of nearby heteroatoms, halogenation can lead to metabolic vulnerabilities due to heterolytic cleavage of the C–X bond by substitution or elimination.^82,81*a*^ This can lead to the formation of free fluoride ion and subsequent toxic effects.

**Fig. 26:**
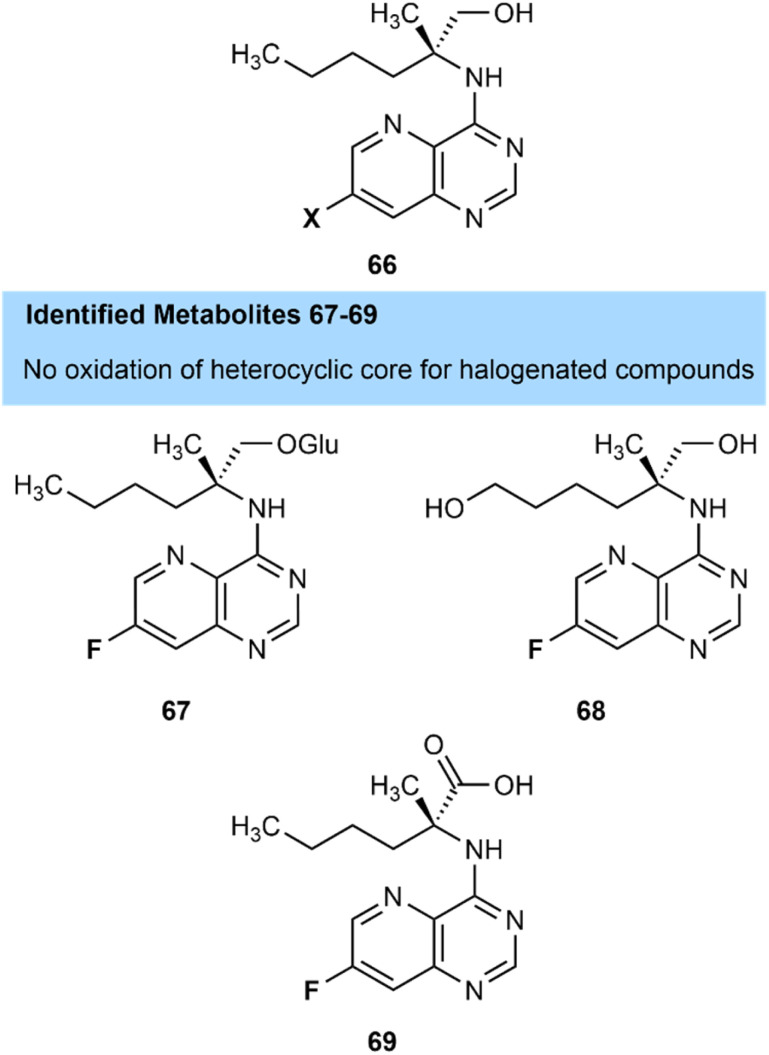
Identified metabolites of selgantolimod, demonstrating halogenation blocks oxidation of heterocyclic core.

In the development of selgantolimod, a hepatitis B treatment, Mackman and team at Gilead Sciences studied the metabolism of their active fluorinated compound 66, its chlorinated derivative as well as a non-halogenated precursor (Fig. 26).^83^ They showed that the non-halogenated compound suffered from extensive oxidative metabolism of the heterocyclic core and had some concerns that these metabolites may be immunologically active *in vivo*. Pleasingly fluorination of the heterocycle prevented oxidation of the heterocyclic core. However, metabolism was redirected to other metabolic hotspots, and metabolites of the fluorinated compound including glucuronidated 67, side-chain hydroxylated 68 and carboxylic acid 69 derivatives were identified which were shown to have no toxicological concerns. The fluorinated compound had a high first pass clearance rate due to this redirected metabolism. The chlorinated derivative had similar metabolic properties to its fluorinated counterpart, but the fluorinated compound was selected due to its slightly higher activity.

**Fig. 27:**
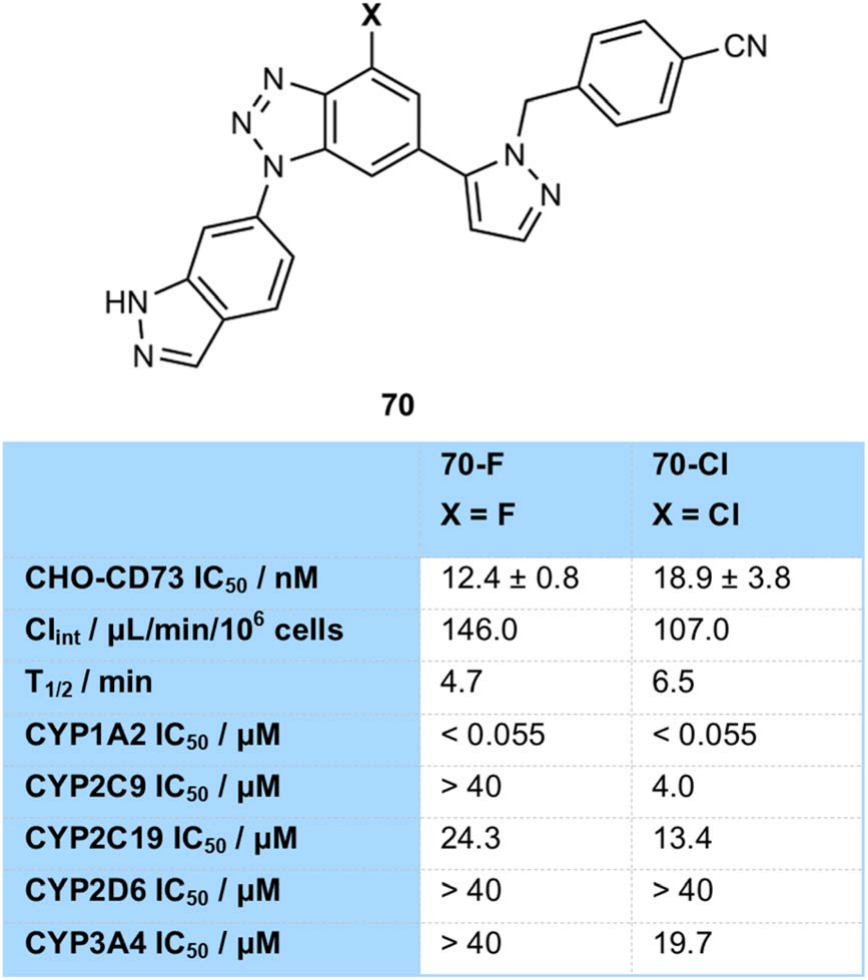
Metabolic properties of halogenated CD73 inhibitors.

Lawson and co-workers developed inhibitors of CD73, which has been shown to be upregulated in tumours and inhibits immune function (Fig. 27).^84^ A fluorinated and chlorinated analogue of their benzotriazole scaffold 70 were prepared, both with similar activity. They showed that the chlorinated compound had improved properties in terms of slower clearance rate and longer half-life (*t*_1/2_70-F = 4.7 min; 70-Cl = 6.5 min), but had more issues in terms of inhibition of CYP enzymes (*e.g.* CYP2C9 IC_50_70-F > 40 µM; 70-Cl = 4 µM) which could lead to potential drug–drug interactions, again showing that choice of halogen is a fine balancing act when it comes to metabolic profiles.

## Conclusion

Overall, this analysis has shown both chlorine and fluorine to be excellent options to include as ring substituents in medicinal chemistry. Chlorinated compounds showed a small but clear improvement in binding constants to targets, that was backed up by chlorine's slight improvement in performance in functional biological assays. However, this slight advantage of chlorine may easily be overcome by other factors in the design and optimization of real drug systems. In general, both chlorine and fluorine showed similar performance in improving bioavailability of molecules. The clearest advantage of fluorine over chlorine was shown in significant improvements in solubility as well as reduced lipophilicity. In addition, whilst the toxicity of fluorinated and chlorinated compounds are generally quite similar, chlorination gives a higher chance of a large increase in toxicity. Both proved to be excellent options to improve bioavailability and block metabolism.

Our work highlights the need to obtain more experimental matched pair data, particularly in pharmacokinetic and metabolic data where little is available and conclusions could not be firm. Often workers only obtain this data on their final most promising compounds, but understanding could be improved if more high-throughput assays were available to test all synthesised compounds quickly. In particular, outlier results both in terms of substrates with significantly higher activity of either halogen (sometimes greater than 100 fold higher activity is observed), or significantly different physicochemical properties, highlight our lack of understanding in many cases of why a seemingly minor structural change leads to significant observed effects. We have also highlighted how good structural evidence such as X-ray crystal structures or quantum mechanics/MD simulations can help us to explain many of these outlier cases. It will also be important to prepare more structurally diverse matched pairs, such as in benzene bioisosteres or aliphatic systems to understand whether information gathered from aromatic and heteroaromatic systems applies to more diverse scaffolds.^85^

We expect that both of these halogen substituents will continue to see significant attention in drug design.

## Author contributions

Connor Summerfield: literature searching and interpretation, production of figures, editing of manuscript. Graham Pattison: data analysis, literature searching and interpretation, production of figures, writing and editing of manuscript.

## Conflicts of interest

There are no conflicts to declare.

## Supplementary Material

SC-017-D5SC07348K-s001

SC-017-D5SC07348K-s002

## Data Availability

The data supporting this article have been included as part of the supplementary information (SI). Supplementary information: spreadsheets containing raw matched-pair data. See DOI: https://doi.org/10.1039/d5sc07348k.

## References

[cit1] (b) Williams R. E. , Leatherwood H. M. and Njardarson J. T., Top 200 Small Molecule Drugs by Sales in 2023, https://bpb-us-e2.wpmucdn.com/sites.arizona.edu/dist/9/130/files/2024/07/2023Top200SmallMoleculePosterV6.pdf, accessed 14/10/2024

[cit2] Smith B. R., Eastman C. M., Njardarson J. T. (2014). J. Med. Chem..

[cit3] Benedetto Tiz D., Bagnoli L., Rosati O., Marini F., Sancineto L., Santi C. (2022). Molecules.

[cit4] Fang W.-Y., Ravindar L., Rakesh K. P., Manukumar H. M., Shantharam C. S., Alharbi N. S., Qin H.-L. (2019). Eur. J. Med. Chem..

[cit5] Purser S., Moore P. R., Swallow S., Gouverneur V. (2008). Chem. Soc. Rev..

[cit6] Sheridan R. P., Hunt P., Culberson J. C. (2006). J. Chem. Inf. Model..

[cit7] Topliss J. G. (1972). J. Med. Chem..

[cit8] Davies M., Nowotka M., Papadatos G., Dedman N., Gaulton A., Atkinson F., Bellis L., Overington J. P. (2015). Nucleic Acids Res..

[cit9] O'Hagan D. (2008). Chem. Soc. Rev..

[cit10] Nelson Jr R. D. , Lide D. R. and Maryott A. A., Selected Values of electric dipole moments for molecules in the gas phase, U.S Department of Commerce, 1967

[cit11] CRC Handbook of Chemistry and Physics, CRC Press/Taylor & Francis, 103rd edn (Internet Version), 2022

[cit12] Bondi A. (1964). J. Phys. Chem..

[cit13] Schlosser M. (1998). Angew. Chem., Int. Ed..

[cit14] Schwerdtfeger P., Nagle J. K. (2019). Mol. Phys..

[cit15] Johnson T. W., Gallego R. A., Edwards M. P. (2018). J. Med. Chem..

[cit16] Shaikh N. S., Iyer J. P., Munot Y. S., Mukhopadhyay P. P., Raje A. A., Nagaraj R., Jamdar V., Gavhane R., Lohote M., Sherkar P., Bala M., Petla R., Meru A., Umrani D., Rouduri S., Joshi S., Reddy S., Kandikere V., Bhuniya D., Kulkarni B., Mookhtiar K. A. (2019). Bioorg. Med. Chem. Lett..

[cit17] Becherer J. D., Boros E. E., Carpenter T. Y., Cowan D. J., Deaton D. N., Haffner C. D., Jeune M. R., Kaldor I. W., Poole J. C., Preugschat F., Rheault T. R., Schulte C. A., Shearer B. G., Shearer T. W., Shewchuk L. M., Smalley Jr T. L., Stewart E. L., Stuart J. D., Ulrich J. C. (2015). J. Med. Chem..

[cit18] Woo S. Y., Kim J. H., Moon M. K., Han S.-H., Yeon S. K., Choi J. W., Jang B. K., Song H. J., Kang Y. G., Kim J. W., Lee J., Kim D. J., Hwang O., Park K. D. (2014). J. Med. Chem..

[cit19] Choi J. W., Kim S., Park J.-H., Kim H. J., Shin S. J., Kim J. W., Woo S. Y., Lee C., Han S. M., Lee J., Pae A. N., Han G., Park K. D. (2019). J. Med. Chem..

[cit20] Waring M. J. (2010). Expert Opin. Drug Discovery.

[cit21] Hasumi K., Sato S., Saito T., Kato J.-y., Shirota K., Sato J., Suzuki H., Ohta S. (2014). Bioorg. Med. Chem..

[cit22] Strong K. L., Epplin M. P., Bacsa J., Butch C. J., Burger P. B., Menaldino D. S., Traynelis S. F., Liotta D. C. (2017). J. Med. Chem..

[cit23] Wenthur C. J., Gentry P. R., Mathews T. P., Lindsley C. W. (2014). Annu. Rev. Pharmacol. Toxicol..

[cit24] Huang H., Degnan A. P., Balakrishnan A., Easton A., Gulianello M., Huang Y., Matchett M., Mattson G., Miller R., Santone K. S., Senapati A., Shields E. E., Sivarao D. V., Snyder L. B., Westphal R., Whiterock V. J., Yang F., Bronson J. J., Macor J. E. (2016). Bioorg. Med. Chem. Lett..

[cit25] Jeffrey Conn P., Christopoulos A., Lindsley C. W. (2009). Nat. Rev. Drug Discovery.

[cit26] Adams D. R., Tawati S., Berretta G., Rivas P. L., Baiget J., Jiang Z., Alsfouk A., Mackay S. P., Pyne N. J., Pyne S. (2019). J. Med. Chem..

[cit27] Clark T., Hennemann M., Murray J. S., Politzer P. (2007). J. Mol. Model..

[cit28] Drinkwater N., Vu H., Lovell K. M., Criscione K. R., Collins B. M., Prisinzano T. E., Poulsen S.-A., McLeish M. J., Grunewald G. L., Martin J. L. (2010). Biochem. J..

[cit29] Partyka A., Kurczab R., Canale V., Satała G., Marciniec K., Pasierb A., Jastrzębska-Więsek M., Pawłowski M., Wesołowska A., Bojarski A. J., Zajdel P. (2017). Bioorg. Med. Chem..

[cit30] Kupcewicz B., Malecka M., Zapadka M., Krajewska U., Rozalski M., Budzisz E. (2016). Bioorg. Med. Chem. Lett..

[cit31] Kupcewicz B., Małecka M. (2015). Cryst. Growth Des..

[cit32] Wang L., Casey M. C., Vernekar S. K. V., Sahani R. L., Kankanala J., Kirby K. A., Du H., Hachiya A., Zhang H., Tedbury P. R., Xie J., Sarafianos S. G., Wang Z. (2020). Eur. J. Med. Chem..

[cit33] Wylie A. A., Schoepfer J., Jahnke W., Cowan-Jacob S. W., Loo A., Furet P., Marzinzik A. L., Pelle X., Donovan J., Zhu W., Buonamici S., Hassan A. Q., Lombardo F., Iyer V., Palmer M., Berellini G., Dodd S., Thohan S., Bitter H., Branford S., Ross D. M., Hughes T. P., Petruzzelli L., Vanasse K. G., Warmuth M., Hofmann F., Keen N. J., Sellers W. R. (2017). Nature.

[cit34] Sessler C. D., Rahm M., Becker S., Goldberg J. M., Wang F., Lippard S. J. (2017). J. Am. Chem. Soc..

[cit35] Dunitz J. D., Taylor R. (1997). Chem. - Eur. J..

[cit36] Howard J. A. K., Hoy V. J., O'Hagan D., Smith G. T. (1996). Tetrahedron.

[cit37] Kovács A., Varga Z. (2006). Coord. Chem. Rev..

[cit38] Bourassa-Bataille H., Sauvageau P., Sandorfy C. (1963). Can. J. Chem..

[cit39] Abraham M. H., Abraham R. J., Aliev A. E., Tormena C. F. (2015). Phys. Chem. Chem. Phys..

[cit40] Wolters L. P., Bickelhaupt F. M. (2012). ChemistryOpen.

[cit41] Chen X., Kopecky D. J., Mihalic J., Jeffries S., Min X., Heath J., Deignan J., Lai S., Fu Z., Guimaraes C., Shen S., Li S., Johnstone S., Thibault S., Xu H., Cardozo M., Shen W., Walker N., Kayser F., Wang Z. (2012). J. Med. Chem..

[cit42] Kumar S., Waldo J. P., Jaipuri F. A., Marcinowicz A., Van Allen C., Adams J., Kesharwani T., Zhang X., Metz R., Oh A. J., Harris S. F., Mautino M. R. (2019). J. Med. Chem..

[cit43] Lee K. S. S., Ng J. C., Yang J., Hwang S. H., Morisseau C., Wagner K., Hammock B. D. (2020). Bioorg. Med. Chem..

[cit44] Qiu Z., Lin X., Zhang W., Zhou M., Guo L., Kocer B., Wu G., Zhang Z., Liu H., Shi H., Kou B., Hu T., Hu Y., Huang M., Yan S. F., Xu Z., Zhou Z., Qin N., Wang Y. F., Ren S., Qiu H., Zhang Y., Zhang Y., Wu X., Sun K., Zhong S., Xie J., Ottaviani G., Zhou Y., Zhu L., Tian X., Shi L., Shen F., Mao Y., Zhou X., Gao L., Young J. A. T., Wu J. Z., Yang G., Mayweg A. V., Shen H. C., Tang G., Zhu W. (2017). J. Med. Chem..

[cit45] Lin X., Shi H., Zhang W., Qiu Z., Zhou Z., Dey F., Zhong S., Qiu H., Xie J., Zhou X., Yang G., Tang G., Shen H. C., Zhu W. (2019). J. Med. Chem..

[cit46] Nam G., Jung J. M., Park H. J., Baek S. Y., Baek K. S., Mok H. Y., Kim D. E., Jung Y. H. (2019). Bioorg. Med. Chem..

[cit47] Olsen J. A., Banner D. W., Seiler P., Obst Sander U., D'Arcy A., Stihle M., Müller K., Diederich F. (2003). Angew. Chem., Int. Ed..

[cit48] Groom C. R., Bruno I. J., Lightfoot M. P., Ward S. C. (2016). Acta Crystallogr., Sect. B.

[cit49] Olsen J., Seiler P., Wagner B., Fischer H., Tschopp T., Obst-Sander U., Banner D. W., Kansy M., Müller K., Diederich F. (2004). Org. Biomol. Chem..

[cit50] Cormanich R. A., Rittner R., O'Hagan D., Bühl M. (2016). J. Comput. Chem..

[cit51] Alleyne C., Amin R. P., Bhatt B., Bianchi E., Blain J. C., Boyer N., Branca D., Embrey M. W., Ha S. N., Jette K., Johns D. G., Kerekes A. D., Koeplinger K. A., LaPlaca D., Li N., Murphy B., Orth P., Ricardo A., Salowe S., Seyb K., Shahripour A., Stringer J. R., Sun Y., Tracy R., Wu C., Xiong Y., Youm H., Zokian H. J., Tucker T. J. (2020). J. Med. Chem..

[cit52] Tucker T. J., Embrey M. W., Alleyne C., Amin R. P., Bass A., Bhatt B., Bianchi E., Branca D., Bueters T., Buist N., Ha S. N., Hafey M., He H., Higgins J., Johns D. G., Kerekes A. D., Koeplinger K. A., Kuethe J. T., Li N., Murphy B., Orth P., Salowe S., Shahripour A., Tracy R., Wang W., Wu C., Xiong Y., Zokian H. J., Wood H. B., Walji A. (2021). J. Med. Chem..

[cit53] Zhang D., Blanco M. J., Ying B. P., Kohlman D., Liang S. X., Victor F., Chen Q., Krushinski J., Filla S. A., Hudziak K. J., Mathes B. M., Cohen M. P., Zacherl D., Nelson D. L., Wainscott D. B., Nutter S. E., Gough W. H., Schaus J. M., Xu Y. C. (2015). Bioorg. Med. Chem. Lett..

[cit54] Axford J., Sung M. J., Manchester J., Chin D., Jain M., Shin Y., Dix I., Hamann L. G., Cheung A. K., Sivasankaran R., Briner K., Dales N. A., Hurley B. (2021). J. Med. Chem..

[cit55] O'Hagan D. (2012). J. Org. Chem..

[cit56] Akkerman F., Buschmann J., Lentz D., Luger P., Rödel E. (2003). J. Chem. Crystallogr..

[cit57] Martins F. A., Freitas M. P. (2019). Eur. J. Org Chem..

[cit58] Abraham R. J., Kemp R. H. (1971). J. Chem. Soc. B.

[cit59] Rodrigues Silva D., de Azevedo Santos L., Hamlin T. A., Fonseca Guerra C., Freitas M. P., Bickelhaupt F. M. (2021). ChemPhysChem.

[cit60] Baranac-Stojanović M. (2014). RSC Adv..

[cit61] Lau C., Anitole K., Hodes C., Lai D., Pfahles-Hutchens A., Seed J. (2007). Toxicol. Sci..

[cit62] Safe S. H. (1994). Crit. Rev. Toxicol..

[cit63] Mackay D., Fraser A. (2000). Environ. Pollut..

[cit64] Nawrozkij M. B., Forgione M., Yablokov A. S., Lucidi A., Tomaselli D., Patsilinakos A., Panella C., Hailu G. S., Kirillov I. A., Badia R., Riveira-Muñoz E., Crespan E., Armijos Rivera J. I., Cirilli R., Ragno R., Esté J. A., Maga G., Mai A., Rotili D. (2019). J. Med. Chem..

[cit65] Wang L., Edwards T. C., Sahani R. L., Xie J., Aihara H., Geraghty R. J., Wang Z. (2021). Eur. J. Med. Chem..

[cit66] Fujita T., Iwasa J., Hansch C. (1964). J. Am. Chem. Soc..

[cit67] Kung P.-P., Rui E., Bergqvist S., Bingham P., Braganza J., Collins M., Cui M., Diehl W., Dinh D., Fan C., Fantin V. R., Gukasyan H. J., Hu W., Huang B., Kephart S., Krivacic C., Kumpf R. A., Li G., Maegley K. A., McAlpine I., Nguyen L., Ninkovic S., Ornelas M., Ryskin M., Scales S., Sutton S., Tatlock J., Verhelle D., Wang F., Wells P., Wythes M., Yamazaki S., Yip B., Yu X., Zehnder L., Zhang W.-G., Rollins R. A., Edwards M. (2016). J. Med. Chem..

[cit68] Glyn R. J., Pattison G. (2021). J. Med. Chem..

[cit69] Böhm H.-J., Banner D., Bendels S., Kansy M., Kuhn B., Müller K., Obst-Sander U., Stahl M. (2004). ChemBioChem.

[cit70] Lapointe G., Skepper C. K., Holder L. M., Armstrong D., Bellamacina C., Blais J., Bussiere D., Bian J., Cepura C., Chan H., Dean C. R., De Pascale G., Dhumale B., Fisher L. M., Fulsunder M., Kantariya B., Kim J., King S., Kossy L., Kulkarni U., Lakshman J., Leeds J. A., Ling X., Lvov A., Ma S., Malekar S., McKenney D., Mergo W., Metzger L., Mhaske K., Moser H. E., Mostafavi M., Namballa S., Noeske J., Osborne C., Patel A., Patel D., Patel T., Piechon P., Polyakov V., Prajapati K., Prosen K. R., Reck F., Richie D. L., Sanderson M. R., Satasia S., Savani B., Selvarajah J., Sethuraman V., Shu W., Tashiro K., Thompson K. V., Vaarla K., Vala L., Veselkov D. A., Vo J., Vora B., Wagner T., Wedel L., Williams S. L., Yendluri S., Yue Q., Yifru A., Zhang Y., Rivkin A. (2021). J. Med. Chem..

[cit71] Cioffi C. L., Racz B., Varadi A., Freeman E. E., Conlon M. P., Chen P., Zhu L., Kitchen D. B., Barnes K. D., Martin W. H., Pearson P. G., Johnson G., Blaner W. S., Petrukhin K. (2019). J. Med. Chem..

[cit72] Hammill J. T., Scott D. C., Min J., Connelly M. C., Holbrook G., Zhu F., Matheny A., Yang L., Singh B., Schulman B. A., Guy R. K. (2018). J. Med. Chem..

[cit73] Degnan A. P., Chaturvedula P. V., Conway C. M., Cook D. A., Davis C. D., Denton R., Han X., Macci R., Mathias N. R., Moench P., Pin S. S., Ren S. X., Schartman R., Signor L. J., Thalody G., Widmann K. A., Xu C., Macor J. E., Dubowchik G. M. (2008). J. Med. Chem..

[cit74] van der Westhuyzen R., Winks S., Wilson C. R., Boyle G. A., Gessner R. K., Soares de Melo C., Taylor D., de Kock C., Njoroge M., Brunschwig C., Lawrence N., Rao S. P. S., Sirgel F., van Helden P., Seldon R., Moosa A., Warner D. F., Arista L., Manjunatha U. H., Smith P. W., Street L. J., Chibale K. (2015). J. Med. Chem..

[cit75] Hruby V. J., Bartosz-Bechowski H., Davis P., Slaninova J., Zalewska T., Stropova D., Porreca F., Yamamura H. I. (1997). J. Med. Chem..

[cit76] Pinto D. J. P., Orwat M. J., Wang S., Fevig J. M., Quan M. L., Amparo E., Cacciola J., Rossi K. A., Alexander R. S., Smallwood A. M., Luettgen J. M., Liang L., Aungst B. J., Wright M. R., Knabb R. M., Wong P. C., Wexler R. R., Lam P. Y. S. (2001). J. Med. Chem..

[cit77] Hirose M., Okaniwa M., Miyazaki T., Imada T., Ohashi T., Tanaka Y., Arita T., Yabuki M., Kawamoto T., Tsutsumi S., Sumita A., Takagi T., Sang B.-C., Yano J., Aertgeerts K., Yoshida S., Ishikawa T. (2012). Bioorg. Med. Chem..

[cit78] Velcicky J., Schlapbach A., Heng R., Revesz L., Pflieger D., Blum E., Hawtin S., Huppertz C., Feifel R., Hersperger R. (2018). ACS Med. Chem. Lett..

[cit79] Chopra D., Guru Row T. N. (2008). CrystEngComm.

[cit80] Shou W. Z. (2020). J. Pharm. Anal..

[cit81] Pan Y. (2019). ACS Med. Chem. Lett..

[cit82] Tuan E., Kirk K. L. (2006). J. Fluorine Chem..

[cit83] Mackman R. L., Mish M., Chin G., Perry J. K., Appleby T., Aktoudianakis V., Metobo S., Pyun P., Niu C., Daffis S., Yu H., Zheng J., Villasenor A. G., Zablocki J., Chamberlain J., Jin H., Lee G., Suekawa-Pirrone K., Santos R., Delaney W. E. I. V., Fletcher S. P. (2020). J. Med. Chem..

[cit84] Beatty J. W., Lindsey E. A., Thomas-Tran R., Debien L., Mandal D., Jeffrey J. L., Tran A. T., Fournier J., Jacob S. D., Yan X., Drew S. L., Ginn E., Chen A., Pham A. T., Zhao S., Jin L., Young S. W., Walker N. P., Leleti M. R., Moschütz S., Sträter N., Powers J. P., Lawson K. V. (2020). J. Med. Chem..

[cit85] Lovering F., Bikker J., Humblet C. (2009). J. Med. Chem..

