# Supplementary material for: Which halogen to choose? Comparing the effects of chlorine and fluorine as bioisosteric substituents in drug design
Source: Chem Sci. 2026 Jan 8;17(5):2477–505. doi: 10.1039/d5sc07348k (PMC12814216; doi:10.1039/d5sc07348k)
Supplement: SC-017-D5SC07348K-s001 [file SC-017-D5SC07348K-s001.pdf]

Supplementary Information (SI) for Chemical Science.
This journal is © The Royal Society of Chemistry 2026

Matched Pair data final.xlsx
This file cannot be rendered in this PDF. Please download the source file.
